# Lumbar Muscle Morphology Correlates With Early Surgical Outcomes in Adolescent Idiopathic Scoliosis: A Pilot Study

**DOI:** 10.1177/21925682261461523

**Published:** 2026-07-01

**Authors:** Paolo Brigato, Sergio De Salvatore, Davide Palombi, Leonardo Oggiano, Camilla Ravaioli, Michele Inverso, Lorenzo Maria Gregori, Andrea Magistrelli, Gianluca Vadalà, Rocco Papalia, Lisbet Haglund, Jean Albert Ouellet, Pier Francesco Costici

**Affiliations:** 1 Research Unit of Orthopaedic and Trauma Surgery, Department of Medicine and Surgery, Università Campus Bio-Medico di Roma, Roma, Italy; 2 Department of Orthopedics and Traumatology Unit, Fondazione Policlinico Universitario Campus Bio-Medico, Roma, Italy; 3 Orthopedic and Traumatology Unit, Department of Surgery, Bambino Gesù Children’s Hospital, Rome, Italy; 4 University Institute for Spine Surgery, Armand Trousseau Hospital, Sorbonne University, 75012 Paris, France; 5 Orthopedics and Traumatology Unit, Department of Geriatrics, Neurosciences and Orthopedics, 18654 Fondazione Policlinico Universitario A. Gemelli IRCCS, Rome, Italy; 6 Diagnostic Imaging Unit, Bambino Gesù Children’s Hospital, IRCCS, Rome, Italy; 7 Orthopaedic Research Laboratory, Department of Orthopedic Surgery, 5620 McGill University, Montreal, QC, Canada; 8 McGill Scoliosis and Spine Group, Department of Surgery, 5620 McGill University, Montreal, QC, Canada

**Keywords:** adolescent idiopathic scoliosis, AIS, paraspinal muscles, iliopsoas, muscle fatty infiltration, cross-sectional area, CSA, magnetic resonance imaging, postoperative recovery, outcomes

## Abstract

**Study Design:**

Original Research.

**Objectives:**

This study aimed to evaluate the preoperative cross-sectional area (CSA) and muscle quality of the major lumbar muscles in patients with adolescent idiopathic scoliosis (AIS) and to investigate their associations with perioperative outcomes. The study also explored lumbar muscle morphology as a potential imaging-based surrogate marker for perioperative risk stratification.

**Materials and Methods:**

In this retrospective study, 81 consecutive AIS patients who underwent posterior spinal fusion at a single institution between January 2019 and December 2023 were included. Two trained pediatric radiologists independently reviewed preoperative T1-weighted axial MRI scans at the L3 level to assess muscle CSA, Modified Goutallier grade, and threshold-based parameters of fatty infiltration. Interobserver reliability was evaluated using intraclass correlation coefficients. Multivariable linear regression analyses were performed to assess associations between muscle parameters and perioperative outcomes, adjusting for relevant covariates. Receiver operating characteristic (ROC) analysis was used to identify curve-specific muscle thresholds for perioperative risk stratification (*P* < .05).

**Results:**

Patients were stratified according to Lenke type: 1-2 (Group 1, n = 30), 5 (Group 2, n = 14), and 3-4-6 (Group 3, n = 37). Fatty infiltration of the multifidus and erector spinae muscles was significantly higher in Groups 2 and 3 compared with Group 1 (*P* < .05). Associations between muscle morphology and perioperative outcomes were curve-pattern dependent, with no significant correlations in Group 1. In Group 2, larger functional CSA of the multifidus, paraspinal muscles, and quadratus lumborum was independently associated with increased blood loss (β = 98.06, *P* = .001; β = 17.49, *P* = .017; β = 29.3, *P* = .014). Greater psoas fatty infiltration predicted delayed ambulation (β = 0.236, *P* = .005). In Group 3, increased paraspinal fatty degeneration predicted longer operative duration (β = 8.42, *P* = .015). ROC analysis identified curve-specific MRI-derived thresholds for perioperative risk stratification.

**Conclusion:**

Preoperative lumbar muscle quality, rather than muscle size, was associated with perioperative complexity and recovery in AIS surgery in a curve-pattern–dependent manner. MRI-based muscle assessment may represent a potential tool for perioperative risk stratification, although prospective validation is required.

## Introduction

Surgical correction of adolescent idiopathic scoliosis (AIS) represents one of the most demanding procedures in spine surgery, characterized by long operative times, substantial blood loss, and potential perioperative complications.^1,2,3,4^ In this setting, accurate preoperative assessment is crucial for optimizing outcomes and minimizing morbidity.^5,6^ Traditionally, predictive factors have included demographic, radiographic, and clinical variables.^6,7,8^ Recently, however, increasing attention has focused on muscle morphology and quality, particularly the cross-sectional area (CSA) and fatty degeneration of paraspinal and hip flexor muscles, as potential markers of physical reserve and postoperative recovery capacity.^9,10,11,12,13^

In adult spine surgery, sarcopenia and fatty muscle degeneration have been increasingly recognized as indicators of poor physiological status and adverse surgical outcomes.^14,15,16,17,18,19^ Several studies have reported that reduced psoas or paraspinal CSA and increased fatty infiltration correlate with longer hospital stay, higher complication rates, and delayed functional recovery after spinal procedures.^14,15,17,18,19^ These findings underscore the increasing clinical significance of muscle health as a component of preoperative risk stratification.

However, to the best of our knowledge, the prognostic role of muscle morphology has not yet been systematically investigated in pediatric populations, particularly in patients with AIS. The adolescent musculoskeletal system differs markedly from that of adults in its adaptive and regenerative potential, making direct extrapolation of adult data unreliable.^20^ In AIS, asymmetric loading and chronic postural imbalance may lead to selective atrophy or fatty infiltration of paraspinal muscles, especially on the concave side of the curve.^20,21,22^ Whether these preoperative muscular alterations influence perioperative parameters, such as blood loss, operative time, or early recovery, remains largely unexplored.

This study aims to investigate the relationship between preoperative lumbar muscle morphology, including CSA and fatty infiltration, and early postoperative outcomes in patients with AIS undergoing posterior spinal fusion (PSF). Additionally, it was evaluated whether these muscular characteristics differ across Lenke curve types and whether they have specific implications for surgical and short-term recovery metrics. By clarifying these associations, this study aims to determine whether preoperative lumbar muscle quality assessment may serve as a predictive marker of perioperative performance and function as an imaging-based surrogate marker for perioperative risk stratification in pediatric spinal deformity surgery.

## Materials and Methods

### Patient Population

The study cohort consisted of consecutive pediatric patients with AIS who underwent elective PSF between January 2019 and December 2023 at a single center. Two experienced senior surgeons performed all procedures. Prior to surgery, each patient underwent thorough pulmonary and cardiovascular assessments and received medical clearance. All patients had a minimum follow-up duration of 2 years.

### Study Design

This retrospective observational cohort study was conducted in accordance with the Strengthening the Reporting of Observational Studies in Epidemiology (STROBE) guidelines.^23^ As part of routine preoperative assessment, all patients scheduled for surgical correction underwent magnetic resonance imaging (MRI) to screen for potential intraspinal abnormalities. Preoperative T1-weighted axial lumbar spine MRI scans acquired preoperatively were retrospectively reviewed. Muscle morphometry was evaluated by analyzing both quantitative (muscle size) and qualitative (muscle composition) parameters bilaterally for several muscle groups: the posterior paraspinal muscles (erector spinae and multifidus), the anterior paravertebral muscle (psoas), and the lateral paravertebral muscle (quadratus lumborum). T1-weighted sequences were preferred over T2-weighted imaging due to their superior ability to differentiate muscle tissue from fatty infiltration, thereby enhancing the accuracy of muscle morphology assessment.^24,25^ Measurements were performed at the level of the inferior endplate of L3. The L3 vertebral level was selected as the standardized reference point, as it has been consistently demonstrated to provide a reliable and anatomically reproducible landmark for cross-sectional assessment of lumbar musculature in imaging-based morphometric analyses.^26,27^ Both radiologists were blinded to all clinical and perioperative outcome data at the time of image analysis.

Inclusion criteria were: (1) confirmed diagnosis of AIS; (2) age between 10 and 21 years; (3) availability of pre-operative T1-weighted MRI including the L3 vertebral level; and (4) minimum clinical follow-up of 24 months. Exclusion criteria included non-idiopathic etiologies, a history of previous spinal surgery, missing data, MRI studies affected by artifacts that precluded accurate muscle segmentation, imaging examinations that did not extend to the L3 vertebral level, MRI examinations performed at external institutions, as well as patients who experienced postoperative complications associated with a prolonged postoperative course, who were excluded to minimize potential confounding effects on early outcome measures.

Patients were categorized according to their curve pattern using the Lenke classification.^1,2,3^ Based on this, they were grouped into three categories: thoracic curves only (Group 1, Lenke 1 and 2), lumbar curves only (Group 2, Lenke 5), and double/triple curves (Group 3, Lenke 3, 4, and 6).

The primary objective of this study was to investigate the association between preoperative lumbar muscle morphology and early postoperative surgical outcomes in patients with AIS undergoing PSF, as well as to determine whether such associations vary across specific curve subtypes. A further objective was to identify clinically meaningful, MRI-based muscle cut-off values associated with increased perioperative risk, thereby exploring the potential role of preoperative lumbar muscle quality assessment as a predictive marker for perioperative performance in pediatric spinal deformity surgery.

### Magnetic Resonance Imaging (MRI) Scan Protocol

Lumbar spine MRI examinations were obtained according to the routine institutional imaging protocol using 1.5-T Magnetom Sola systems (Siemens Healthineers, Germany), without any protocol customization for the purposes of this study. The standard imaging protocol comprised sagittal T1-and T2-weighted sequences covering the lumbosacral spine from T12 to S2, along with axial acquisitions extending from L1 through S1. Representative sequence parameters were as follows: sagittal T2-weighted imaging (TR/TE ≈ 3500/90 ms, slice thickness 4 mm); sagittal T1-weighted imaging (TR/TE ≈ 450-699/10-20 ms, slice thickness 4 mm); axial T2-weighted imaging (TR/TE ≈ 4000/90 ms, slice thickness 4 mm); and sagittal STIR sequences (TR/TE ≈ 3500/90 ms). Total acquisition time ranged from approximately 10 to 16 minutes.

### Postoperative Protocol

All patients underwent an opioid-sparing analgesic regimen. Early mobilization was encouraged, with patients assisted to sit after the first 24 hours and to stand and ambulate with assistance after the first 48 hours following surgery. The subfascial drain and urinary catheter were removed 48 hours postoperatively. Antibiotic prophylaxis with intravenous cefazolin was administered intraoperatively and continued for 48 hours postoperatively. A standing anteroposterior and lateral spinal radiograph was performed 48 hours after ambulation to verify correction and implant position.

### Study Variables and Outcome Measures

#### Study Variables

Preoperative data collected included age, sex, American Society of Anesthesiologists (ASA) Score, Lenke type, Body Mass Index (BMI) and Risser Stage.

**Figure 1.**
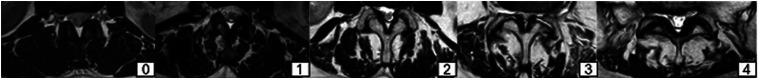
Example of T2-weighted axial images showing the grading of fatty infiltration of paraspinal muscles according to the Modified Goutallier Classification (MGC). Grade 0: No fatty infiltration, Grade 1: Few fatty streaks within the muscles, Grade 2: Less than 50% fat within the muscle, Grade 3: 50% of fat within the muscles, Grade 4: More than 50% fat within the muscle. Reproduced from Mandelli et al.^28^ under the Creative Commons Attribution License (CC BY), Copyright © 2021 Mandelli, Nüesch, Zhang, Halbeisen, Schären, Mündermann and Netzer

The morphology of the main lumbar muscles was characterized using a combination of quantitative and qualitative metrics. These included the CSA, Psoas Muscle Index (PMI), Psoas–Vertebral Ratio (PVR), Paraspinal-Vertebral Ratio (PPVR), Paraspinal Muscle Index (PPMI), Psoas–Paraspinal Muscle Ratio (PPMR), Modified Goutallier Classification grade (MGC), Fat fraction (%Fat), fat area (FA), functional cross-sectional area percentage (%FCSA) and functional cross-sectional area (FCSA) (Table 1). For each muscle, quantitative and qualitative muscle metrics were measured on both the concave and convex sides of each curve, and the mean value was used for analysis. This approach was chosen to reflect the overall muscular condition of the patient rather than side-specific adaptation, as overall muscle reserve is considered the primary determinant of physiological perioperative response. Furthermore, given the well-documented variability in concave-convex asymmetry across different Lenke curve types, using mean values allowed for consistent, reproducible comparisons across subgroups. The other indices were calculated from CSA and patient characteristics to provide normalized and relative measures of muscle size. CSA was measured on axial MRI slices using ImageJ software (National Institutes of Health, Bethesda, MD, USA) by manually tracing regions of interest (ROIs) encompassing the psoas, erector spinae, multifidus, and quadratus lumborum muscles. FA and FCSA were calculated from the %Fat and %FCSA, respectively, providing quantitative estimates of the absolute extent of fatty infiltration and the remaining contractile muscle component. Muscle fatty infiltration was also qualitatively assessed for each muscle using the MGC (Figure 1).^28,29^

**Table 1.** Muscle parameters and measurement methods

Muscle parameter	Definition/Formula	Notes
CSA (Cross-Sectional Area)	Area of the muscle measured on preoperative axial MRI	Quantitative measurement performed using ImageJ (National Institutes of Health, Bethesda, MD, USA). Applied to psoas, erector spinae, multifidus, and quadratus lumborum.
PMI (Psoas Muscle Index)	(CSA of right + left psoas)/(height in meters)^2	Normalizes psoas size to body size
PVR (Psoas-Vertebral Ratio)	(CSA of right + left psoas)/(CSA of corresponding vertebral body)	Normalizes psoas size to vertebral body size
PPMI (Paraspinal Muscle Index)	(CSA of right + left paraspinal muscles)/(height in meters)^2	Normalizes paraspinal muscles size to body size
PPVR (Paraspinal-Vertebral Ratio)	(CSA of right + left paraspinal muscles)/(CSA of corresponding vertebral body)	Normalizes paraspinal muscles size to vertebral body size
PPMR (Psoas-Paraspinal Muscle Ratio)	(CSA of right + left psoas)/(CSA of paraspinal muscles)	Provides relative size of psoas compared to paraspinal musculature
GC (Goutallier Classification)	Visual grading of fatty degeneration.	Applied to psoas, erector spinae, multifidus, and quadratus lumborum muscles (Grade 0: no fatty infiltration; Grade 1: few fatty streaks within the muscle; Grade 2: fat occupying <50% of the muscle; Grade 3: fat occupying approximately 50% of the muscle; Grade 4: fat occupying >50% of the muscle)
%Fat (Fat Fraction)	(% of intramuscular fat within the muscle CSA)	Represents the proportion (%) of fatty tissue relative to total muscle CSA. Quantitative measurement performed using ImageJ (National Institutes of Health, Bethesda, MD, USA). Applied to psoas, erector spinae, multifidus, and quadratus lumborum.
FA (Fat Area)	FA = CSA × (%Fat/100)	Quantitative parameter representing the absolute intramuscular fat content (cm^2^).
%FCSA (Functional Cross-Sectional Area Percentage)	%FCSA = 100 − %Fat	Represents the proportion (%) of contractile (non-fatty) muscle tissue relative to total muscle CSA.
FCSA (Functional Cross-Sectional Area)	FCSA = CSA − Fat Area	Quantitative parameter representing the absolute contractile component of the muscle (cm^2^).

CSA: Cross-Sectional Area; PMI: Psoas Muscle Index; PPMI: Paraspinal Muscle Index; PVR: Psoas-Vertebral Ratio; PPVR: Paraspinal-Vertebral Ratio; PPMR: Psoas-Paraspinal Muscle Ratio; GC: Goutallier Classification; %Fat: Intramuscular Fat Fraction; FA: Fat Area; %FCSA: Functional Cross-Sectional Area Percentage; FCSA: Functional Cross-Sectional Area; MRI: Magnetic Resonance Imaging.

Radiographic assessments included preoperative measurements of main and minor curve magnitudes (Cobb angles) and curve flexibility (%), as well as postoperative measurements of main and minor curves with calculation of corresponding correction rates (CRs). Instrumentation data collected comprised the number of fused levels, total pedicle screws and number of pedicle screws per level. Surgical outcomes included estimated blood loss (EBL, ml), length of hospital stay (LOS, days), surgery duration (min), and time to assisted ambulation(days). EBL was estimated by the anesthesia team based on suction volume and sponge weight.

**Figure 2.**
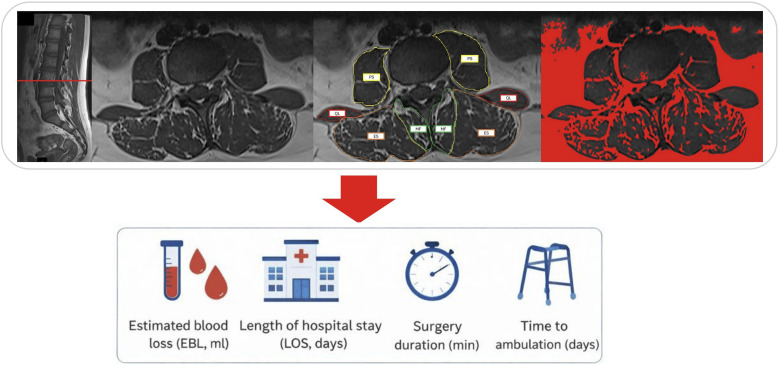
Example of lumbar muscle segmentation, quantitative MRI analysis, and associated clinical outcomes. Upper panel (from left to right): (A) sagittal T1-weighted MRI centered on the L3 vertebral body, indicating the level of the axial slice; (B) native axial T1-weighted image acquired at the inferior endplate of L3; (C) manual region-of-interest (ROI) segmentation of lumbar muscles performed using ImageJ, including bilateral psoas (PS), erector spinae (ES), multifidus (MF), and quadratus lumborum (QL); (D) threshold-based segmentation map highlighting fatty infiltration (FI), used to calculate fat percentage (%Fat) and derive quantitative parameters such as fat area (FA) and functional cross-sectional area (FCSA). Lower panel: schematic representation of the main perioperative outcomes analyzed in the study, including estimated blood loss (EBL, ml), length of hospital stay (LOS, days), surgery duration (min), and time to assisted ambulation (days)

Figure 2 shows ImageJ-based ROI segmentation and threshold-based %Fat computation on axial T1-weighted MRI at the inferior endplate of L3, along with the main clinical outcomes of interest.

#### Outcome Measures

Curve flexibility of the major and minor curves, which was considered the main predictor of curve stiffness, was calculated as follows:

• ((Preop static major/minor curve-Preop major/minor curve on bending films)/Preop static major/minor curve)*100.

The postoperative CR of the major and minor curves were calculated as follows:

• ((Preop static major/minor curve-Post-halo/Postop/Last FUP major/minor curve)/Preop static major/minor curve)*100.

#### Interobserver Reliability

All muscle measurements were performed by two trained pediatric radiologists. Interobserver reliability for continuous variables (CSA and %Fat) was assessed using the intraclass correlation coefficient (ICC [2,1], two-way random-effects model with absolute agreement), with values of 0.75-0.90 indicating good agreement and ≥0.90 indicating excellent agreement.^30^ Agreement for MGC was evaluated using quadratic weighted Cohen’s kappa. Results are reported with 95% confidence intervals (CIs), and only paired measurements with complete data from both observers were analyzed.

### Statistical Analysis and Ethics

Data analysis was performed using RStudio version 4.4.3. Continuous variables are reported as mean ± standard deviation (SD) and range, and normality was assessed using the Shapiro–Wilk test. Between-group comparisons were conducted using one-way ANOVA for normally distributed variables and the Kruskal–Wallis test for non-normally distributed variables. Categorical variables were compared using the chi-square test or Fisher’s exact test, as appropriate. Associations between preoperative paraspinal muscle characteristics and perioperative outcomes were examined using a two-step approach. Univariate relationships were initially assessed with Spearman’s correlation analysis, after which multivariable linear regression models were constructed to identify independent predictors within each Lenke group. Models were adjusted for age, BMI, and surgery duration (included as a covariate to isolate muscle effects from surgical complexity). Multicollinearity was checked using the Variance Inflation Factor (VIF), ensuring no redundant variables were included (VIF < 5). An exploratory clinical threshold analysis was performed to identify actionable “risk profiles” using receiver operating characteristic (ROC) curve analysis. “High-risk” events were defined as outcome values above the 75th percentile of the respective group distribution. For each muscle-related predictor, the optimal cut-off value was determined using the Youden index to maximize the combined sensitivity and specificity. Statistical significance was set at *P* < .05.

The study was conducted in accordance with international ethical guidelines for clinical research, as outlined in the Helsinki and Istanbul Declarations. As this study involved retrospective analysis of existing medical records with no patient intervention or alteration of clinical management, formal IRB approval was not required, and waived in accordance with institutional policy.

## Results

### Patient and Group Selection

**Figure 3.**
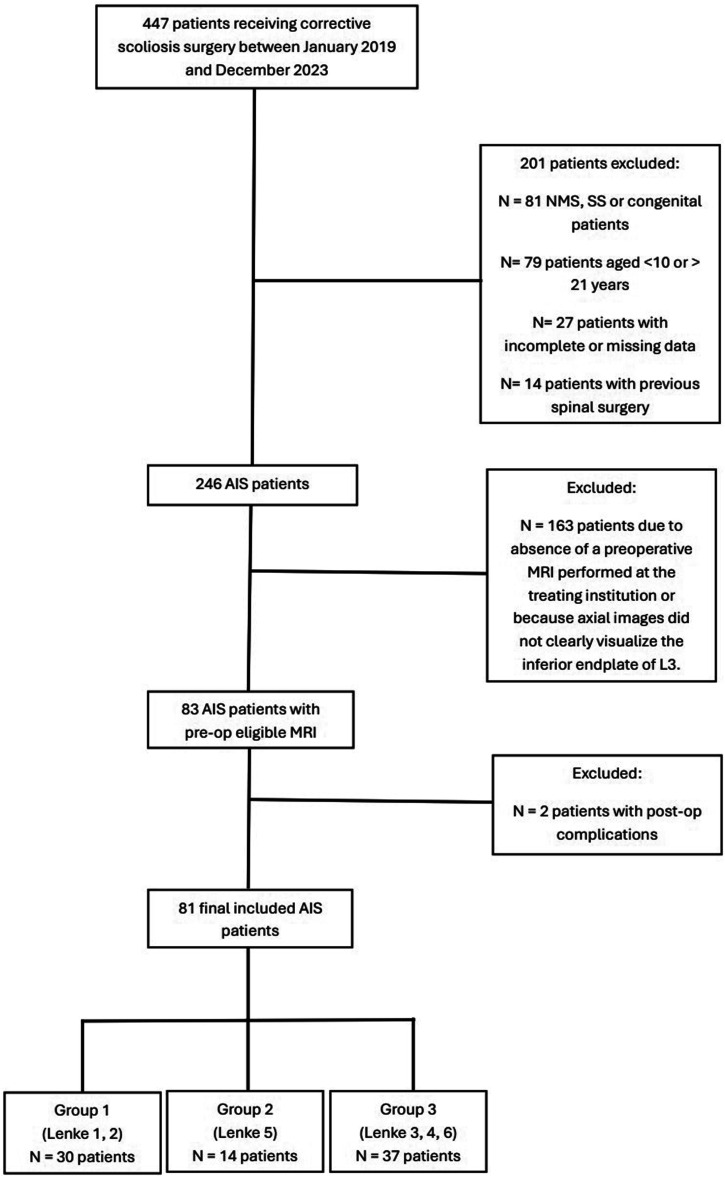
Patients’ selection

Between January 2019 and December 2023, 447 pediatric patients underwent surgical correction. Of these, 201 were excluded due to neuromuscular, congenital, or syndromic etiology, two-stage procedures, missing data, or age outside the inclusion range, leaving 246 AIS patients who underwent single-stage PSF. In-site preoperative MRI was available 83 patients. Two additional patients were excluded due to postoperative complications (one superficial infection and one dural tear), both of which considerably prolonged the LOS. Therefore, the final study cohort comprised 81 AIS patients. These were further stratified into Group 1 (Lenke 1-2, n = 30), Group 2 (Lenke 5, n = 14), and Group 3 (Lenke 3-4-6, n = 37). The patient selection process is summarized in Figure 3.

### Demographic and Anthropometric Characteristics

The cohort was predominantly female across all groups (73.3% in Group 1, 92.9% in Group 2, and 83.8% in Group 3). Most patients were classified as ASA 1 in all groups, with ASA 2 representing a minority and only one ASA 3 patient in Group 3. Group 1 included Lenke type 1 (66.7%) and type 2 (33.3%) curves, whereas Group 3 was mainly composed of Lenke type 3 curves (54.1%), followed by types 6 (32.4%) and 4 (13.5%) (Table 2). There were no statistically significant differences in mean age, BMI and Risser stage among the groups, ensuring comparability of the baseline characteristics (Table 3).

**Table 2.** Demographic Characteristics

Variable (N, %)	Group 1 (N = 30)	Group 2 (N = 14)	Group 3 (N = 37)
Sex			
M	8 (26.7)	1 (7.1)	6 (16.2)
F	22 (73.3)	13 (92.9)	31 (83.8)
ASA score			
1	26 (86.7)	10 (71.4)	26 (70.3)
2	4 (13.3)	4 (28.6)	10 (27.0)
3	/	/	1 (2.7)
Lenke type			
Type 1	20 (66.7)	/	/
Type 2	10 (33.3)	/	/
Type 3	/	/	20 (54.1)
Type 4	/	/	5 (13.5)
Type 5	/	14 (100.0)	/
Type 6	/	/	12 (32.4)

ASA: American Society of Anesthesiologists Physical Status Classification; F: Female; M: Male.

**Table 3.** Anthropometric Characteristics

Variable	Group 1 (N = 30)	Group 2 (N = 14)	Group 3 (N = 37)	*P*-value
Age (years)	14.73 ± 1.62 (12.00-18.00)	14.14 ± 1.56 (12.00-17.00)	15.08 ± 1.77 (12.00-19.00)	0.192
BMI (kg/m2)	20.23 ± 2.71 (17.21-30.11)	20.91 ± 4.11 (15.85-32.60)	21.80 ± 4.29 (15.57-35.14)	0.255
Risser stage	3.9 ± 0.76 (2-5)	3.7 ± 1.2 (2-5)	3.7 ± 0.95 (2-5)	0.884

BMI: Body Mass Index. Data are presented as mean ± standard deviation (range). *P*-values were computed using the Kruskal–Wallis test.

### Interobserver Reliability

Interobserver reliability is reported in Table 4. Agreement for CSA and %Fat measurements was moderate to excellent across muscle groups, with highest reliability for psoas and erector spinae CSA. In contrast, MGC showed lower and more variable agreement, particularly for the psoas and quadratus lumborum muscles.

**Table 4.** Interobserver Reliability for Muscle Measurements (Intraclass Correlation Coefficient/Quadratic Weighted Cohen’s Kappa)

Measure	n	Reliability metric	Value (95% CI)	Interpretation
Multifidus				
CSA	81	ICC	0.65 (0.38-0.79)	Moderate
%Fat	81	ICC	0.75 (0.50-0.83)	Good
GC	81	κw	0.53 (0.22-0.77)	Moderate
PSOAS				
CSA	81	ICC	0.96 (0.68-0.98)	Excellent
%Fat	81	ICC	0.80 (0.52-0.93)	Good
GC	81	κw	0.39 (0.15-0.58)	Poor
Quadratus lomborum				
CSA	81	ICC	0.67 (0.53-0.78)	Moderate
%Fat	81	ICC	0.76 (0.60-0.79)	Good
GC	81	κw	0.20 (0.06-0.44)	Poor
Erector spinae				
CSA	81	ICC	0.90 (0.79-0.96)	Excellent
%Fat	81	ICC	0.87 (0.69-0.95)	Good
GC	81	κw	0.63 (0.14-0.82)	Moderate

ICC: intraclass correlation coefficient (two-way random-effects model, absolute agreement, single measurements); κw: quadratic weighted Cohen’s kappa. CSA: cross-sectional area; GC: Goutallier classification.

### Muscle Morphology Characteristics

Preoperative paraspinal muscle characteristics are reported in Table 5. No significant differences were identified among groups with respect to psoas muscle parameters (all *P* > .05). Likewise, no between-group differences were observed for erector spinae, multifidus, total paraspinal or quadratus lumborum CSA (all *P* > .05). In contrast, significant group-related differences emerged in muscle quality measures. MGC of the erector spinae, multifidus, and overall paraspinal musculature were consistently higher in Group 3 compared with the other groups (*P* < .05). Additionally, Group 3 demonstrated more advanced quantitative degeneration of the multifidus and paraspinal muscles, as reflected by significantly higher %Fat and FA values and lower %FCSA (*P* < .05). Overall, a clear gradient of progressive paraspinal muscle degeneration was observed across groups, with Group 1 < Group 2 < Group 3.

**Table 5.** Preoperative Muscle Characteristics

Parameters	Group 1 (N = 30)	Group 2 (N = 14)	Group 3 (N = 37)	*P*
Psoas muscles				
Psoas CSA (mean) (mean, SD, range)	7.7 (±3.0) (3.3-14.1)	8.2 (±2.3) (3.9-13.2)	8.7 (±2.7) (3.9-14.5)	0.488
Psoas-to-vertebral body ratio (mean, SD, range)	0.7 (±0.2) (0.3-1.2)	0.8 (±0.2) (0.4-1.2)	0.8 (±0.2) (0.4-1.4)	0.188
PMI (mean, SD, range)	2.9 (±0.9) (1.4-4.5)	3.1 (±0.7) (1.7-4.4)	3.5 (±1.0) (1.8-5.8)	0.142
Psoas Goutallier grade (mean) (mean, SD, range)	1.1 (±0.4) (0.5-2.2)	1.1 (±0.4) (0.5-2.2)	1.2 (±0.4) (0.5-2.2)	0.183
Psoas fat percentage (mean) (mean, SD, range)	4.7 (±3.8) (0.2-12.9)	2.6 (±2.6) (0.1-8.5)	3.1 (±3.0) (0.1-12.5)	0.080
Psoas fat area (mean) (mean, SD, range)	0.3 (±0.3) (0.0-1.3)	0.2 (±0.2) (0.0-0.5)	0.3 (±0.3) (0.0-1.1)	0.166
Psoas FCSA percentage (mean) (mean, SD, range)	95.3 (±3.8) (87.1-99.8)	97.4 (±2.6) (91.5-99.9)	96.9 (±3.0) (87.6-99.9)	0.076
Psoas FCSA area (mean) (mean, SD, range)	7.4 (±3.0) (3.1-13.9)	8.0 (±2.3) (3.7-13.2)	8.4 (±2.7) (3.7-13.9)	0.418
Erector spinae muscles				
Erector Spinae CSA (mean) (mean, SD, range)	13.4 (±4.4) (7.3-22.1)	11.4 (±3.6) (7.5-18.3)	13.0 (±2.8) (8.1-20.9)	0.132
Erector Spinae Goutallier grade (mean) (mean, SD, range)	1.4 (±0.4) (0.5-2.2)	1.7 (±0.5) (1.0-2.5)	1.9 (±0.6) (1.0-3.5)	<0.05*
Erector Spinae fat percentage (mean) (mean, SD, range)	15.3 (±6.2) (2.7-30.4)	18.1 (±8.1) (5.8-28.4)	20.8 (±9.8) (1.2-46.0)	0.055
Erector Spinae fat area (mean) (mean, SD, range)	2.1 (±1.2) (0.1-5.2)	1.8 (±0.6) (0.9-2.7)	2.7 (±1.6) (0.2-6.8)	0.112
Erector Spinae FCSA percentage (mean) (mean, SD, range)	84.7 (±6.2) (69.6-97.3)	81.9 (±8.1) (71.7-94.2)	79.2 (±9.8) (54.0-98.8)	0.058
Erector Spinae FCSA area (mean) (mean, SD, range)	11.3 (±3.8) (6.7-19.6)	9.6 (±3.9) (5.5-17.3)	10.3 (±2.4) (5.0-16.8)	0.139
Multifidus muscles				
Multifidus CSA (mean) (mean, SD, range)	3.8 (±1.1) (2.0-6.6)	3.9 (±1.3) (1.8-5.6)	3.9 (±1.7) (1.5-10.9)	0.872
Multifidus Goutallier grade (mean) (mean, SD, range)	1.6 (±0.5) (1.0-2.5)	2.0 (±0.7) (1.0-3.2)	2.2 (±0.6) (1.2-3.5)	<0.05*
Multifidus fat percentage (mean) (mean, SD, range)	28.0 (±8.6) (9.7-43.7)	35.4 (±9.9) (21.9-51.7)	36.9 (±12.2) (0.4-65.9)	<0.05*
Multifidus fat area (mean) (mean, SD, range)	1.1 (±0.4) (0.3-2.1)	1.4 (±0.6) (0.4-2.5)	1.4 (±0.8) (0.0-4.3)	<0.05*
Multifidus FCSA percentage (mean) (mean, SD, range)	72.0 (±8.6) (56.3-90.3)	64.6 (±9.9) (48.3-78.2)	63.1 (±12.2) (34.1-99.6)	<0.05*
Multifidus FCSA area (mean) (mean, SD, range)	2.7 (±0.9) (1.3-5.2)	2.5 (±0.9) (0.9-3.7)	2.5 (±1.1) (0.6-6.6)	0.330
Paraspinal muscles (multifidus, erector spinae)				
Paraspinal CSA (mean) (mean, SD, range)	17.2 (±5.0) (9.6-27.5)	15.4 (±4.4) (9.8-23.5)	16.9 (±3.7) (10.4-25.4)	0.409
Paraspinal-to-vertebral body ratio (mean, SD, range)	1.6 (±0.5) (0.8-2.7)	1.4 (±0.5) (1.0-2.4)	1.7 (±0.4) (1.0-2.9)	0.121
Paraspinal muscle index (mean, SD, range)	6.4 (±1.6) (3.7-10.1)	5.9 (±1.6) (3.7-9.3)	6.8 (±1.2) (4.5-9.4)	0.119
Psoas-to-paraspinal muscle ratio (mean, SD, range)	0.5 (±0.1) (0.2-0.8)	0.6 (±0.2) (0.4-1.1)	0.5 (±0.2) (0.3-0.9)	0.114
Paraspinal Goutallier grade (mean) (mean, SD, range)	1.4 (±0.4) (0.7-2.2)	1.8 (±0.5) (1.0-2.5)	2.0 (±0.6) (1.1-3.2)	<0.05*
Paraspinal fat percentage (mean) (mean, SD, range)	21.6 (±6.8) (6.5-37.0)	26.8 (±8.1) (16.7-40.0)	28.9 (±9.8) (0.8-51.9)	<0.05*
Paraspinal fat area (mean) (mean, SD, range)	3.8 (±1.7) (1.0-7.8)	4.0 (±1.0) (1.7-5.7)	4.9 (±2.1) (0.2-11.4)	<0.05*
Paraspinal FCSA percentage (mean) (mean, SD, range)	78.4 (±6.8) (63.0-93.5)	73.2 (±8.1) (60.0-83.3)	71.1 (±9.8) (48.1-99.2)	<0.05*
Paraspinal FCSA area (mean) (mean, SD, range)	13.4 (±4.1) (7.6-23.4)	11.4 (±4.2) (7.1-19.4)	12.0 (±2.9) (5.1-18.3)	0.173
Quadratus lumborum muscles				
Quadratus lumborum CSA (mean) (mean, SD, range)	2.7 (±1.3) (1.0-5.8)	3.5 (±2.8) (1.0-12.4)	3.1 (±1.5) (0.9-7.9)	0.444
Quadratus lumborum goutallier grade (mean) (mean, SD, range)	0.9 (±0.3) (0.5-1.8)	1.1 (±0.3) (0.5-1.8)	1.0 (±0.4) (0.5-2.0)	0.234
Quadratus lumborum fat percentage (mean) (mean, SD, range)	4.7 (±3.7) (0.2-13.7)	5.8 (±6.2) (0.2-21.1)	5.9 (±5.0) (0.2-26.3)	0.524
Quadratus lumborum fat area (mean) (mean, SD, range)	0.1 (±0.1) (0.0-0.6)	0.2 (±0.2) (0.0-0.5)	0.2 (±0.2) (0.0-1.1)	0.244
Quadratus lumborum FCSA percentage (mean) (mean, SD, range)	95.3 (±3.7) (86.3-99.8)	94.2 (±6.2) (78.9-99.8)	94.1 (±5.0) (73.7-99.8)	0.522
Quadratus lumborum FCSA area (mean) (mean, SD, range)	2.6 (±1.2) (0.9-5.5)	3.4 (±2.9) (1.0-12.4)	2.9 (±1.3) (0.8-7.3)	0.560

CSA: Cross-sectional area; GC: Goutallier classification; PMI: Psoas muscle index; FCSA: fat cross-sectional area; PVR: psoas-to-vertebral body ratio. Data are presented as mean ± standard deviation (range). *P*-values were computed using the one-way ANOVA or Kruskal–Wallis tests.

### Radiographic Outcomes

Radiographic characteristics are summarized in Table 6. Preoperatively, the main curve magnitude was significantly higher in Group 3 (71.2° ± 16.7) compared with Group 1 (57° ± 12.6) and 2 (52.1° ± 1.8) (*P* < .0001). Main curve flexibility was also significantly lower in Group 3 (20.9% ± 15.7) than in Group 1 (28.8% ± 14.1) and Group 2 (48.1% ± 26.4) (*P* < .05). Postoperatively, the main curve magnitude remained higher in Group 3 (22.8° ± 11.2) compared with Group 1 (14.2° ± 5.7) and Group 2 (12.4° ± 5.6) (*P* < .05). Similarly, the main curve CR was lower in Group 3 (68.6% ± 10.1) than in Group 1 (75.8% ± 7.1) and Group 2 (76.1% ± 11.1) (*P* < .05). For Group 3, minor curve magnitude and flexibility were 54.8° ± 13.7 and 28.1% ± 14.5, respectively, with a postoperative minor curve CR of 69.8% ± 14.1.

**Table 6.** Radiographic Characteristics

Parameters	Group 1 (N = 30)	Group 2 (N = 14)	Group 3 (N = 37)	*P*
Preoperative				
Main curve magnitude (°, mean, SD, range)	57.0 (±12.6) (40.0-82.0)	52.1 (±1.8) (50.0-55.0)	71.2 (±16.7) (45.0-104.0)	<0.0001*
Main curve flexibility (%, mean, SD, range)	28.8 (±14.1) (3.7-63.3)	48.1 (±26.4) (3.8-83.3)	20.9 (±15.7) (2.0-54.5)	<0.05*
Minor curve magnitude (°, mean, SD, range)	42.2 (±8.8) (33.0-59.0)	/	54.8 (±13.7) (31.0-86.0)	/
Minor curve flexibility (%, mean, SD, range)	16.9 (±6.8) (7.7-27.3)	/	28.1 (±14.5) (4.2-59.7)	/
Postoperative				
Main curve magnitude (°, mean, SD, range)	14.2 (±5.7) (4-25)	12.4 (±5.6) (7-25)	22.8 (±11.2) (6-60)	<0.05*
Main curve CR (%, mean, SD, range)	75.8 (±7.1) (66.6-91.1)	76.1 (±11.1) (50.9-86.8)	68.6 (±10.1) (42.3-89.1)	<0.05*
Minor curve magnitude (°, mean, SD, range)	10.2 (±3.8) (5-16)	/	16.6 (±9.5) (3-45)	/
Minor curve CR (%, mean, SD, range)	76.1 (±6.8) (60-86.1)	/	69.8 (±14.1) (39.5-94.9)	/

CR: Correction rate. Data are presented as mean ± standard deviation (range). *P*-values were computed using the one-way ANOVA or Kruskal–Wallis tests.

### Operative and Inpatient Data

Surgical and perioperative characteristics are summarized in Table 7. The number of fused levels was significantly higher in Group 3 (13.9 ± 1.5) compared with Group 1 (13 ± 1.1) and Group 2 (10.2 ± 2.8) (*P* < .0001). Similarly, the total number of pedicle screws was greater in Group 3 (23.3 ± 3.5) than in Group 1 (21.1 ± 3.8) and Group 2 (16.5 ± 4.1) (*P* < .05), while the number of screws per level did not differ significantly among groups (*P* = .536). Mean surgery duration differed significantly (*P* < .05), being shortest in Group 2 (223.4 ± 48.9 min) and longer in Group 1 (251.6 ± 34.8 min) and Group 3 (259.2 ± 44.3 min). A similar pattern was observed for intraoperative EBL, which was lowest in Group 2 (362.9 ± 160.5 mL) and highest in Group 3 (637.3 ± 314.3 mL; *p* < .05). Time to ambulation did not differ significantly between groups (approximately 3 days in all groups; *p* = 0.775). In contrast, LOS increased significantly across groups (*p* < .05), from 6.6 ± 0.8 days in Group 1 to 7.7 ± 1.4 days in Group 3, with Group 2 showing intermediate values (7.2 ± 0.8 days).

**Table 7.** Surgical and Hospitalization Data

Parameters	Group 1 (N = 30)	Group 2 (N = 14)	Group 3 (N = 37)	*P*
Number of fused levels (n, mean, SD, range)	13 (±1.1) (10-15)	10.2 (±2.8) (7-14)	13.9 (±1.5) (6-15)	<0.0001*
Number of pedicle screws (n, mean, SD, range)	21.1 (±3.8) (14-29)	16.5 (±4.1) (12-26)	23.3 (±3.5) (12-30)	<0.05*
Number of screws per level (n, mean, SD, range)	1.6 (±0.2) (1.2-2)	1.6 (±0.3) (1.1-2)	1.6 (±0.2) (1.2-2)	0.536
Surgery duration (min, mean, SD, range)	251.6 (±34.8) (190.0-334.0)	223.4 (±48.9) (144.0-321.0)	259.2 (±44.3) (179.0-374.0)	<0.05*
EBL (mL, mean, SD, range)	561.7 (±262.2) (200.0-1200.0)	362.9 (±160.5) (200.0-800.0)	637.3 (±314.3) (200.0-1450.0)	<0.05*
Time to first ambulation (days, mean, SD, range)	3.0 (±0.9) (2.0-4.0)	2.9 (±0.8) (2.0-4.0)	3.1 (±0.7) (2.0-4.0)	0.775
LOS (days, mean, SD, range)	6.6 (±0.8) (5.0-8.0)	7.2 (±0.8) (5.0-8.0)	7.7 (±1.4) (6.0-12.0)	<0.05*

LOS: Length of stay; EBL: Estimated Blood Loss. Data are presented as mean ± standard deviation (range). *P*-values were computed using the one-way ANOVA or Kruskal–Wallis tests.

### Muscle Morphology and Perioperative Outcomes Correlation Analysis

**Figure 4.**
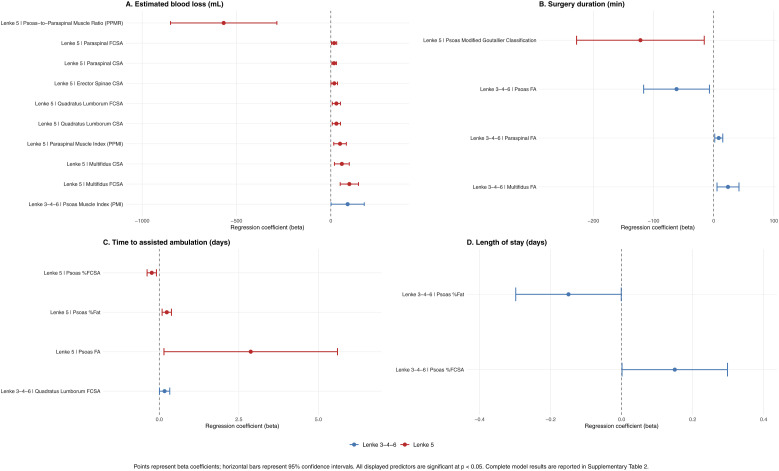
Significant multivariable linear regression predictors of perioperative outcomes stratified by Lenke subgroup. Forest plots show statistically significant associations between lumbar muscle morphology parameters and estimated blood loss, surgery duration, time to assisted ambulation, and length of hospital stay. Points represent β coefficients and horizontal bars represent 95% confidence intervals. Red markers indicate Lenke 5 curves and blue markers indicate Lenke 3-4-6 curves. Only predictors with *P* < 0.05 are shown for clarity; complete multivariable model results, including beta coefficients, standard errors, 95% confidence intervals, exact *P*-values, and model sample sizes, are reported in Supplementary Table 2

Detailed results of the univariate Spearman correlation analyses are reported in Supplemental Table 1, whereas multivariable linear regression analyses are reported in Supplemental Table 2. In Supplementary Table 2, regression coefficients are presented with standard errors, 95% confidence intervals, exact p-values, and model sample sizes. Significant multivariable associations are summarized in Figure 4.

#### Univariate Correlation Analysis

Univariate analysis showed limited, group-specific associations between muscle parameters and perioperative outcomes. In Group 2, muscle quality metrics were associated with surgery duration, blood loss, and time to ambulation (*P* < .05), while in Group 3 significant correlations were mainly restricted to psoas fat–related parameters. No associations regarding Group 1 were found.

#### Multivariable Correlation Analysis of Independent Predictors

##### Group 1: Thoracic Curves (Lenke 1-2)

In patients with non-structural lumbar curves (Lenke 1-2), neither muscle size nor muscle quality parameters were associated with perioperative outcomes, including EBL, LOS, or operative time (all *P* > .05).

##### Group 2: Thoracolumbar/Lumbar Curves (Lenke 5)

In patients with single structural lumbar curves (Lenke 5), posterior muscle mass and anterior muscle quality independently predicted perioperative outcomes. Higher multifidus, paraspinal and quadratus lumborum FCSA areas (β = 98.06 (*P* = .001), β = 17.49 (*P* = .017) and β = 29.3 (*P* = .014), respectively) and PPMI (β = 48.70, *P* = .009) were associated with increased EBL, whereas a higher PPMR predicted reduced EBL (β = −567.98, *P* = .001). Increased psoas %Fat was associated with delayed ambulation (β = 0.236, *P* = .005), whereas higher psoas %FCSA was associated with shorter time to ambulation (β = −0.236, *P* = .005).

##### Group 3: Complex Curves (Lenke 3-4-6)

In patients with double or triple curves (Lenke 3-4-6), associations between posterior muscle degeneration, somatic constitution, and surgical burden were more heterogeneous. Greater fatty infiltration of the posterior musculature was independently associated with prolonged operative duration, with both paraspinal FA (β = 8.42, *P* = .015) and multifidus FA (β = 23.80, *P* = .012) emerging as significant predictors. Additionally, the PMI was positively associated with intraoperative blood loss (β = 89.08, *P* = .048).

### Clinical Threshold Analysis

**Figure 5.**
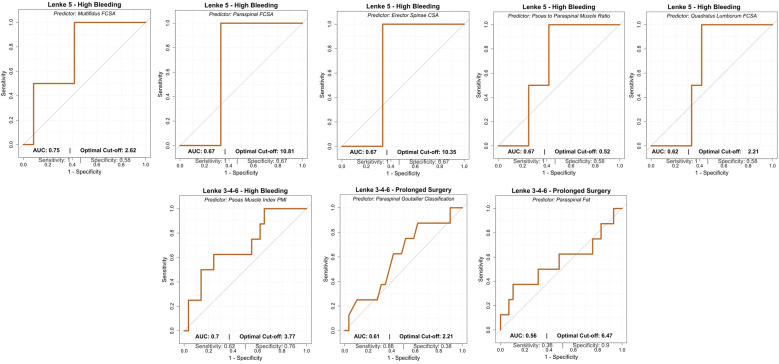
Receiver operating characteristic (ROC) curves for muscle-based predictors of high bleeding and prolonged surgery across Lenke subtypes

ROC analysis identified clinically actionable preoperative cut-off values for perioperative risk stratification. In patients with thoracolumbar/lumbar curves (Lenke 5), posterior muscle hypertrophy demonstrated good discriminatory ability for predicting high intraoperative blood loss (>400 mL), with multifidus FCSA >2.62 cm^2^ (AUC = 0.75), paraspinal FCSA >10.8 cm^2^ (AUC = 0.67), an erector spinae CSA >10.3 cm^2^ (AUC = 0.67), a PPMR <0.52 (AUC = 0.67) and a quadratus lumborum FCSA >2.09 cm^2^ (AUC = 0.62). In patients with complex curves (Lenke 3-4-6), a paraspinal FA >6.47 cm^2^ and a paraspinal MGC >2.21 moderately identified cases with prolonged operative duration (>284 minutes, AUC = 0.56 and AUC = 0.61, respectively), and a PMI >3.77 identified an high risk of bleeding (>800 mL) with an accuracy of 0.70. These findings are shown in Table 8 and summarized in Figure 5.

**Table 8.** Receiver Operating Characteristic (ROC)-Derived Cut-Off Values for Perioperative Risk Stratification and Clinical Interpretation

Lenke group	Outcome (threshold)	Muscle parameter	Cut-off value	AUC (accuracy)	Sensitivity	Specificity	Clinical interpretation interpretation
Lenke 5	High bleeding (>400 mL)	Multifidus FCSA	> 2.62 cm^2^	0.75	1.00	0.58	Greater posterior muscle mass → increased bleeding risk
​	​	Paraspinal FCSA	> 10.81 cm^2^	0.67	1.00	0.67	Greater posterior muscle mass → increased bleeding risk
​	​	Erector spinae CSA	> 10.35 cm^2^	0.67	1.00	0.67	Greater posterior muscle mass → increased bleeding risk
​	​	Psoas-to-paraspinal muscle ratio	< 0.52	0.67	1.00	0.58	Posterior muscle predominance is associated with a higher bleeding risk
​	​	Quadratus lumborum FCSA	> 2.09 cm^2^	0.62	1.00	0.58	Greater posterior muscle mass → increased bleeding risk
Lenke 3-4-6	High bleeding (>800 mL)	Psoas muscle index (PMI)	> 3.77	0.70	0.62	0.76	Higher somatic muscle mass is associated with increased bleeding risk
​	Prolonged surgery (>284 min)	Paraspinal goutallier Grade	> 2.21	0.61	0.88	0.38	Advanced muscle degeneration associated with complex deformity and prolonged surgery
​	​	Paraspinal FA	> 6.47 cm^2^	0.56	0.38	0.90	Advanced muscle degeneration associated with complex deformity and prolonged surgery

ROC: receiver operating characteristic; AUC: area under the curve; FCSA: functional cross-sectional area; CSA: cross-sectional area; FA: fat area; PMI: psoas muscle index. Sensitivity and specificity reported at the optimal Youden index cut-off. High-risk outcomes defined as values above the 75th percentile of the respective group distribution.

## Discussion

This study evaluated the relationship between preoperative lumbar muscle morphology and early postoperative outcomes in AIS patients undergoing PSF, finding that muscle quality, rather than muscle size, was meaningfully associated with early postoperative outcomes. Muscle morphology–outcome relationships were strongly curve-pattern dependent. No associations between lumbar muscle metrics and perioperative outcomes were identified in Group 1 (Lenke 1-2), supporting the anatomical specificity of the analysis when surgical exposure is confined to the thoracic spine. In contrast, patients in Group 2 (Lenke 5) exhibited distinct, independent predictors of perioperative burden. Greater posterior muscle mass, reflected by increased multifidus, paraspinal, and quadratus lumborum FCSA as well as higher posterior PPMI, was independently associated with increased intraoperative blood loss, whereas a higher PPMR exerted a protective effect. Additionally, anterior muscle quality influenced early recovery, as increased psoas fatty infiltration predicted delayed ambulation, while higher psoas %FCSA was associated with earlier mobilization. In complex curve patterns (Lenke 3-4-6), the relationship between muscle morphology and surgical burden was more heterogeneous, likely reflecting the influence of multiple coexisting anatomical and procedural factors inherent to complex curve patterns. Posterior muscle degeneration emerged as the dominant determinant, with greater fatty infiltration of the paraspinal and multifidus muscles independently predicting prolonged operative duration, likely reflecting the increased curve severity and surgical complexity characteristic of these deformity patterns. Furthermore, somatic constitution, as reflected by a higher PMI, was associated with increased intraoperative EBL, underscoring the multifactorial nature of surgical complexity in double- and triple-curve patterns. ROC analysis identified clinically actionable preoperative thresholds for risk stratification. In Lenke 5 curves, posterior muscle hypertrophy reliably discriminated patients at risk for high blood loss (>400 mL), whereas in Lenke 3-4-6 curves, paraspinal fatty degeneration and elevated muscle indices identified patients at increased risk for prolonged operative time (>284 minutes) and excessive bleeding (>800 mL). Overall, these findings suggest that muscle quality, more than muscle size, is a curve-dependent determinant of perioperative complexity and early recovery in AIS surgery. This conclusion requires qualification: in Lenke 5 curves specifically, posterior muscle size (as reflected by FCSA) was itself a significant predictor of intraoperative blood loss, underscoring that the relative importance of muscle size versus quality varies by curve pattern. Across the full cohort, however, qualitative parameters, particularly fatty infiltration, demonstrated broader and more consistent associations with perioperative outcomes.

In adult spinal surgery, sarcopenia and muscle fatty degeneration are well-established predictors of adverse outcomes.^10,14,15^ Studies by Bokshan et al demonstrated that reduced psoas CSA independently predicted longer LOS, higher complication rates, and increased morbidity following thoracolumbar fusion.^15^ Similarly, Hirase et al reported sarcopenia as an independent risk factor for perioperative complications.^16^ Ferrero et al also linked poor paraspinal muscle quality to postoperative imbalance in adult deformity.1^11^ Conversely, McKenzie et al found no significant effect of sarcopenia on outcomes in less-invasive lumbar procedures, suggesting that the clinical impact of muscle loss may depend on surgical complexity and patient frailty status.^17^

When stratified by curve type, our preliminary evidence suggested distinct patterns of muscular involvement. Main thoracic curves (Group 1) showed relatively preserved lumbar muscle morphology and no association with intraoperative metrics or LOS. In contrast, lumbar (Group 2) and double/complex (Group 3) curves were associated with longer LOS (7.2 ± 0.8 and 7.7 ± 1.4 days) compared with Group 1 (6.6 ± 0.8 days, *P* < .05). These findings suggest that lumbar-predominant deformities may impose greater mechanical stress on the psoas and posterior extensor muscles, promoting localized muscle remodeling.

Consistent with findings from adult populations, muscle morphology in our cohort was also associated with EBL and operative time.^13,18,31^ Notably, neither BMI nor age correlated significantly with CSA or fatty infiltration (all *P* > .1), supporting the interpretation that the observed muscular changes are primarily curve-related rather than metabolic or age-dependent.^31^

The greater fatty degeneration observed in Group 2 and 3 curves may result from chronic asymmetric loading and compensatory muscular adaptation in the lumbar region.^20,21,31^ Increased torsional and shear forces in these curves place continuous stress on the multifidus, erector spinae, and psoas muscles, leading to localized disuse atrophy on the concave side and compensatory hypertrophy or fiber-type transformation on the convex side, as described by Stetkarova et al and Becker et al, representing a biomechanical adaptation rather than a degenerative process.^21,31^ This pattern suggests that even subtle, subclinical alterations in muscle morphology may translate into meaningful differences in early postoperative recovery, despite the absence of systemic frailty or sarcopenia. Consistently, in our cohort, Group 2 patients with greater psoas fatty infiltration experienced delayed ambulation, whereas a higher psoas %FCSA was associated with earlier postoperative mobilization.^15,16,18,19^ Interestingly, total muscle CSA did not show a clear correlation with perioperative parameters or LOS, suggesting that muscle quality, rather than quantity, is clinically relevant. This finding aligns with the adult data from Urakawa et al and Bokshan et al, who emphasised fatty infiltration and low attenuation as more sensitive predictors of outcome than CSA alone.^10,15^

The preliminary findings of this study underscore the potential clinical relevance of preoperative muscle assessment in AIS. Although traditional predictors, such as curve magnitude and skeletal maturity, are valuable, MRI-based muscle evaluation may enhance prediction of early postoperative outcomes and could serve as a valid variables in future risk models, similar to the current use of sarcopenia and muscle quality indices in adult spinal deformity surgery.^7,32,33,34^ In this context, our ROC analyses identified curve-specific preoperative muscle cut-off values associated with increased intraoperative EBL and prolonged operative time, suggesting a potential role for muscle morphology in perioperative risk stratification. Nevertheless, these thresholds should be considered exploratory, and larger prospective studies are still required to validate their clinical applicability and generalizability. Furthermore, the use of the 75th percentile to define “high-risk” outcomes, while pragmatic in the context of a small pilot cohort, is inherently arbitrary, and the modest discriminatory ability of these thresholds (AUC range 0.56-0.75) precludes their direct clinical application without further validation. Furthermore, identifying patients with greater muscle fatty infiltration may allow for tailored rehabilitation protocols, earlier physiotherapy, or targeted prehabilitation strategies aimed at optimizing muscle function prior to surgery, as supported by our finding that Lenke 5 patients with higher muscle %Fat experienced a significantly longer time to ambulation.^35^

### Strengths and Limitations

This study presents the first investigation evaluating the relationship between preoperative lumbar muscle morphology and early postoperative outcomes in patients with AIS undergoing PSF. The selection of the L3 vertebral level as a standardized reference point is a methodological strength of this study, as it has consistently provided a reliable, anatomically reproducible landmark for cross-sectional assessment of the lumbar musculature in imaging-based morphometric analyses. Muscle measurements were independently performed by two experienced pediatric radiologists, and interobserver reliability was formally assessed using intraclass correlation coefficients, supporting the robustness of the morphometric analysis. The inclusion of detailed, threshold-based muscle parameters represents a methodological strength of this study, as it provides a quantitative framework for assessing muscle quality in this young population, as demonstrated by the higher interobserver reliability of these measures compared with qualitative muscle assessments (MGC). Furthermore, patients were stratified by Lenke curve type, allowing for curve-specific comparisons rather than pooling heterogeneous deformities. This represents a relevant strength, as most existing studies on paravertebral muscle morphology in AIS predominantly focus on isolated thoracic curves and do not systematically evaluate lumbar or complex curve patterns, thereby limiting the understanding of muscle–deformity interactions across the full spectrum of AIS presentations. Furthermore, excluding patients who experienced early postoperative complications helped mitigate potential confounding bias, allowing recovery outcomes to more accurately reflect physiological and functional differences rather than complication-related effects.

However, some limitations should be acknowledged. First, the retrospective and single-center design limits causal inference and external generalizability. Second, the relatively small sample size reduces statistical power and precludes the inclusion of all potentially relevant confounders in multivariable models in order to avoid model overfitting. In particular, subgroup analyses in Group 2 (n = 14) are limited in statistical power, and the multivariable models in this subgroup should be interpreted with caution given the risk of overfitting.Third, although the L3 vertebral level is a widely validated and commonly used reference, it does not account for vertebral rotation and curve-related anatomical distortion inherent to spinal deformity, which may have affected muscle morphology measurements obtained at a single axial level. Moreover, only early recovery variables were analyzed, without long-term functional or radiographic follow-up. Furthermore, potential confounders for early postoperative recovery, such as preoperative physical activity, nutritional status, and postoperative pain levels, were not evaluated and may have influenced functional outcomes, particularly time to ambulation and length of hospital stay. Importantly, the Modified Goutallier Classification showed lower and more variable interobserver agreement compared with quantitative measures, particularly for the psoas and quadratus lumborum muscles. This reduced reliability represents a notable limitation of qualitative muscle grading and should be considered when interpreting findings based on this parameter. Future studies should prioritize quantitative, threshold-based fat quantification approaches, which demonstrated superior reproducibility in this cohort. Finally, although several studies have emphasized the importance of concave–convex asymmetry in AIS, the present analysis used the mean value of both sides to assess overall muscle quality. This approach was chosen to reflect overall muscular condition rather than side-specific adaptation; however, asymmetry-related features may still hold prognostic relevance and warrant further investigation. Additionally, the Lenke-based stratification employed in this study implicitly controls for the major sources of between-group variation in surgical complexity, including number of fused levels, instrumentation density, and curve magnitude, as these parameters are inherently correlated with curve type by definition. Adding them as covariates within already small subgroups would introduce substantial multicollinearity and increase the risk of overfitting, which informed the decision to limit covariate adjustment to age, BMI, and surgery duration.

## Conclusion

Preoperative lumbar muscle morphology is associated with perioperative complexity and early postoperative recovery in AIS patients undergoing PSF, in a curve-pattern–dependent manner. Muscle quality showed greater clinical relevance than muscle size and influenced operative duration, blood loss and time to ambulation in lumbar and complex curves, while no associations were observed in isolated thoracic deformities. These findings may support the future integration of MRI-based muscle assessment into preoperative planning and risk stratification algorithms for AIS surgery. However, prospective validation in larger, multicenter cohorts is required to confirm these associations and to establish their clinical applicability across different patient populations and surgical settings.

## Supplemental Material

Supplemental Material - Lumbar Muscle Morphology Correlates With Early Surgical Outcomes in Adolescent Idiopathic Scoliosis: A Pilot Study Supplemental Material for Lumbar Muscle Morphology Correlates With Early Surgical Outcomes in Adolescent Idiopathic Scoliosis: A Pilot Study by Paolo Brigato, Sergio De Salvatore, Davide Palombi, Leonardo Oggiano, Camilla Ravaioli, Michele Inverso, Lorenzo Maria Gregori, Andrea Magistrelli, Gianluca Vadalà, Rocco Papalia, Lisbet Haglund, Jean Albert Ouellet, Pier Francesco Costici, in Global Spine Journal

Supplemental Material - Lumbar Muscle Morphology Correlates With Early Surgical Outcomes in Adolescent Idiopathic Scoliosis: A Pilot Study Supplemental Material for Lumbar Muscle Morphology Correlates With Early Surgical Outcomes in Adolescent Idiopathic Scoliosis: A Pilot Study by Paolo Brigato, Sergio De Salvatore, Davide Palombi, Leonardo Oggiano, Camilla Ravaioli, Michele Inverso, Lorenzo Maria Gregori, Andrea Magistrelli, Gianluca Vadalà, Rocco Papalia, Lisbet Haglund, Jean Albert Ouellet, Pier Francesco Costici, in Global Spine Journal

## Data Availability

The datasets used and/or analyzed during the current study are not publicly available due to our policy statement of sharing clinical data only on request but are available from the corresponding author on reasonable request.*

## Appendix

### Abbreviations

AIS: adolescent idiopathic scoliosis
ASA: American Society of Anesthesiologists
BMI: body mass index
CR: correction rate
CSA: cross-sectional area
ES: erector spinae
MGC: Modified Goutallier Classification
LOS: length of stay
MF: multifidus
MRI: magnetic resonance imaging
NMS: neuromuscular scoliosis
PMI: psoas muscle index
PPMR: psoas–paraspinal muscle ratio
PS: psoas major
PSF: posterior spinal fusion
PVR: psoas–vertebral ratio
QL: quadratus lumborum
