# Supplementary material for: Lumbar Muscle Morphology Correlates With Early Surgical Outcomes in Adolescent Idiopathic Scoliosis: A Pilot Study
Source: Global Spine J. 2026 Jul 1:21925682261461523. Online ahead of print. doi: 10.1177/21925682261461523 (PMC13323041; doi:10.1177/21925682261461523)
Supplement: Supplemental Material - Lumbar Muscle Morphology Correlates With Early Surgical Outcomes in Adolescent Idiopathic Scoliosis: A Pilot Study [file sj-pdf-1-gsj-10.1177_21925682261461523.pdf]

Supplementary Table 1. Significant univariate correlations

.

lenke_group	outcome	predictor	spearman_rho	p_value
Lenke 1–2	Blood loss	Multifidus GC	0.465	0.010
Lenke 1–2	Blood loss	Paraspinal GC	0.432	0.017
Lenke 1–2	Blood loss	Quadratus Lumborum FAT area	0.382	0.037
Lenke 1–2	LOS	Psoas GC	0.387	0.034
Lenke 3–4–6	Blood loss	Psoas FAT (%)	-0.352	0.033
Lenke 3–4–6	Blood loss	Psoas FCSA (%)	0.350	0.034
Lenke 3–4–6	LOS	Psoas FAT (%)	-0.463	0.004
Lenke 3–4–6	LOS	Psoas FAT area	-0.426	0.009
Lenke 3–4–6	LOS	Psoas FCSA (%)	0.460	0.004
Lenke 3–4–6	Surgery duration	Psoas FAT (%)	-0.333	0.044
Lenke 3–4–6	Surgery duration	Psoas FAT area	-0.388	0.018
Lenke 3–4–6	Surgery duration	Psoas FCSA (%)	0.331	0.045
Lenke 3–4–6	Time to ambulation	Quadratus Lumborum CSA	0.334	0.043
Lenke 3–4–6	Time to ambulation	Quadratus Lumborum FCSA area	0.357	0.030
Lenke 5	Blood loss	Multifidus CSA	0.604	0.022
Lenke 5	Blood loss	Multifidus FCSA area	0.697	0.006
Lenke 5	Blood loss	Paraspinal CSA	0.546	0.044
Lenke 5	Blood loss	Paraspinal FCSA area	0.568	0.034
Lenke 5	Blood loss	Quadratus Lumborum FAT (%)	-0.599	0.024
Lenke 5	Blood loss	Quadratus Lumborum FCSA (%)	0.599	0.024
Lenke 5	Blood loss	Quadratus Lumborum FCSA area	0.572	0.032
Lenke 5	Surgery duration	Psoas-to-paraspinal ratio	0.662	0.010
Lenke 5	Time to ambulation	Erector Spinae FAT (%)	0.571	0.033
Lenke 5	Time to ambulation	Erector Spinae FAT area	0.568	0.034
Lenke 5	Time to ambulation	Erector Spinae FCSA (%)	-0.571	0.033
Lenke 5	Time to ambulation	Erector Spinae FCSA area	-0.613	0.020
Lenke 5	Time to ambulation	Paraspinal FCSA area	-0.564	0.036
Lenke 5	Time to ambulation	Paraspinal muscle index	-0.613	0.020
Lenke 5	Time to ambulation	Psoas FAT (%)	0.652	0.011
Lenke 5	Time to ambulation	Psoas FAT area	0.634	0.015
Lenke 5	Time to ambulation	Psoas FCSA (%)	-0.652	0.011
