# Supplementary material for: Lumbar Muscle Morphology Correlates With Early Surgical Outcomes in Adolescent Idiopathic Scoliosis: A Pilot Study
Source: Global Spine J. 2026 Jul 1:21925682261461523. Online ahead of print. doi: 10.1177/21925682261461523 (PMC13323041; doi:10.1177/21925682261461523)
Supplement: Supplemental Material - Lumbar Muscle Morphology Correlates With Early Surgical Outcomes in Adolescent Idiopathic Scoliosis: A Pilot Study [file sj-pdf-2-gsj-10.1177_21925682261461523.pdf]

Supplementary Table 2. Multivariable linear regression analyses

Lenke group	Outcome	Predictor	Beta	SE	95% CI	p-value
Group 1 (Lenke 1-2)	Surgery duration (min)	Psoas CSA	0.575	2.470	-4.503 to 5.653	0.81788900
Group 1 (Lenke 1-2)	Surgery duration (min)	Psoas Muscle Index (PMI)	1.619	8.052	-14.932 to 18.170	0.84220200
Group 1 (Lenke 1-2)	Surgery duration (min)	Psoas %Fat	-1.830	1.906	-5.748 to 2.088	0.34593200
Group 1 (Lenke 1-2)	Surgery duration (min)	Psoas FCSA	0.818	2.538	-4.399 to 6.035	0.74980800
Group 1 (Lenke 1-2)	Surgery duration (min)	Psoas PVR	20.344	29.784	-40.877 to 81.565	0.50061400
Group 1 (Lenke 1-2)	Surgery duration (min)	Psoas-to-Paraspinal Muscle Ratio (PPMR)	40.843	52.475	-67.021 to 148.707	0.44340000
Group 1 (Lenke 1-2)	Surgery duration (min)	Psoas Modified Goutallier Classification	-12.875	16.332	-46.446 to 20.697	0.43765100
Group 1 (Lenke 1-2)	Surgery duration (min)	Psoas FA	-15.818	21.840	-60.710 to 29.074	0.47537000
Group 1 (Lenke 1-2)	Surgery duration (min)	Psoas %FCSA	1.830	1.906	-2.088 to 5.748	0.34593200
Group 1 (Lenke 1-2)	Surgery duration (min)	Erector Spinae CSA	-1.011	1.584	-4.266 to 2.244	0.52877300
Group 1 (Lenke 1-2)	Surgery duration (min)	Erector Spinae Modified Goutallier Classification	-2.800	18.010	-39.820 to 34.219	0.87764300
Group 1 (Lenke 1-2)	Surgery duration (min)	Erector Spinae %Fat	-1.222	1.082	-3.446 to 1.001	0.26882400
Group 1 (Lenke 1-2)	Surgery duration (min)	Erector Spinae %FCSA	1.222	1.082	-1.001 to 3.446	0.26882400
Group 1 (Lenke 1-2)	Surgery duration (min)	Erector Spinae FCSA	-0.682	1.848	-4.481 to 3.116	0.71496500
Group 1 (Lenke 1-2)	Surgery duration (min)	Erector Spinae FA	-7.081	5.814	-19.031 to 4.869	0.23417600
Group 1 (Lenke 1-2)	Surgery duration (min)	Multifidus CSA	-2.966	6.565	-16.462 to 10.529	0.65513900
Group 1 (Lenke 1-2)	Surgery duration (min)	Multifidus Modified Goutallier Classification	-10.396	13.792	-38.746 to 17.954	0.45776300
Group 1 (Lenke 1-2)	Surgery duration (min)	Multifidus %Fat	-1.314	0.750	-2.855 to 0.227	0.09138980
Group 1 (Lenke 1-2)	Surgery duration (min)	Multifidus %FCSA	1.314	0.750	-0.227 to 2.855	0.09138980
Group 1 (Lenke 1-2)	Surgery duration (min)	Multifidus FCSA	1.704	7.809	-14.347 to 17.755	0.82896700
Group 1 (Lenke 1-2)	Surgery duration (min)	Multifidus FA	-23.263	14.868	-53.824 to 7.299	0.12976400
Group 1 (Lenke 1-2)	Surgery duration (min)	Paraspinal CSA	-0.931	1.403	-3.814 to 1.952	0.51278900
Group 1	Surgery duration (min)	Paraspinal Muscle Index (PPMI)	-3.538	4.490	-12.768 to	0.43782500

Lenke group	Outcome	Predictor	Beta	SE	95% CI	p-value
(Lenke 1-2)					5.692	
Group 1 (Lenke 1-2)	Surgery duration (min)	Paraspinal %Fat	-1.554	0.956	-3.519 to 0.411	0.11613900
Group 1 (Lenke 1-2)	Surgery duration (min)	Paraspinal FA	-5.409	3.855	-13.332 to 2.514	0.17237400
Group 1 (Lenke 1-2)	Surgery duration (min)	Paraspinal %FCSA	1.554	0.956	-0.411 to 3.519	0.11613900
Group 1 (Lenke 1-2)	Surgery duration (min)	Paraspinal FCSA	-0.382	1.735	-3.948 to 3.184	0.82728100
Group 1 (Lenke 1-2)	Surgery duration (min)	Paraspinal Modified Goutallier Classification	-8.441	17.054	-43.495 to 26.614	0.62479900
Group 1 (Lenke 1-2)	Surgery duration (min)	Quadratus Lumborum CSA	-3.940	5.701	-15.658 to 7.778	0.49560600
Group 1 (Lenke 1-2)	Surgery duration (min)	Quadratus Lumborum %Fat	0.946	2.017	-3.200 to 5.091	0.64303100
Group 1 (Lenke 1-2)	Surgery duration (min)	Quadratus Lumborum %FCSA	-0.946	2.017	-5.091 to 3.200	0.64303100
Group 1 (Lenke 1-2)	Surgery duration (min)	Quadratus Lumborum FCSA	-4.191	5.937	-16.394 to 8.012	0.48645600
Group 1 (Lenke 1-2)	Surgery duration (min)	Quadratus Lumborum Modified Goutallier Classification	9.393	21.102	-33.983 to 52.769	0.65991500
Group 1 (Lenke 1-2)	Surgery duration (min)	Quadratus Lumborum FA	-9.450	63.190	-139.339 to 120.439	0.88227700
Group 1 (Lenke 1-2)	Estimated blood loss / EBL (mL)	Psoas CSA	22.698	17.922	-14.213 to 59.610	0.21701100
Group 1 (Lenke 1-2)	Estimated blood loss / EBL (mL)	Psoas Muscle Index (PMI)	37.975	59.749	-85.080 to 161.029	0.53083000
Group 1 (Lenke 1-2)	Estimated blood loss / EBL (mL)	Psoas %Fat	-10.295	14.595	-40.355 to 19.765	0.48709500
Group 1 (Lenke 1-2)	Estimated blood loss / EBL (mL)	Psoas FCSA	25.219	18.352	-12.578 to 63.015	0.18158300
Group 1 (Lenke 1-2)	Estimated blood loss / EBL (mL)	Psoas PVR	100.260	225.540	-364.248 to 564.768	0.66048200
Group 1 (Lenke 1-2)	Estimated blood loss / EBL (mL)	Psoas-to-Paraspinal Muscle Ratio (PPMR)	147.563	399.949	-676.148 to 971.275	0.71526800
Group 1 (Lenke 1-2)	Estimated blood loss / EBL (mL)	Psoas Modified Goutallier Classification	128.669	122.213	-123.033 to 380.370	0.30249000
Group 1 (Lenke 1-2)	Estimated blood loss / EBL (mL)	Psoas FA	-89.013	165.443	-429.749 to 251.722	0.59531400
Group 1 (Lenke 1-2)	Estimated blood loss / EBL (mL)	Psoas %FCSA	10.295	14.595	-19.765 to 40.355	0.48709500
Group 1 (Lenke 1-2)	Estimated blood loss / EBL (mL)	Erector Spinae CSA	10.509	11.827	-13.849 to 34.866	0.38271400
Group 1 (Lenke 1-2)	Estimated blood loss / EBL (mL)	Erector Spinae Modified Goutallier Classification	67.174	133.957	-208.716 to 343.063	0.62043700
Group 1 (Lenke 1-2)	Estimated blood loss / EBL (mL)	Erector Spinae %Fat	-6.620	8.372	-23.863 to 10.622	0.43652200
Group 1 (Lenke 1-2)	Estimated blood loss / EBL (mL)	Erector Spinae %FCSA	6.620	8.372	-10.622 to 23.863	0.43652200

Lenke group	Outcome	Predictor	Beta	SE	95% CI	p-value
Group 1 (Lenke 1-2)	Estimated blood loss / EBL (mL)	Erector Spinae FCSA	15.911	13.504	-11.900 to 43.722	0.24978100
Group 1 (Lenke 1-2)	Estimated blood loss / EBL (mL)	Erector Spinae FA	-20.716	45.708	-114.853 to 73.422	0.65430200
Group 1 (Lenke 1-2)	Estimated blood loss / EBL (mL)	Multifidus CSA	-15.409	49.321	-116.988 to 86.169	0.75730800
Group 1 (Lenke 1-2)	Estimated blood loss / EBL (mL)	Multifidus Modified Goutallier Classification	171.286	99.525	-33.688 to 376.261	0.09759730
Group 1 (Lenke 1-2)	Estimated blood loss / EBL (mL)	Multifidus %Fat	0.610	6.258	-12.279 to 13.499	0.92314600
Group 1 (Lenke 1-2)	Estimated blood loss / EBL (mL)	Multifidus %FCSA	-0.610	6.258	-13.499 to 12.279	0.92314600
Group 1 (Lenke 1-2)	Estimated blood loss / EBL (mL)	Multifidus FCSA	-19.506	58.294	-139.565 to 100.553	0.74070900
Group 1 (Lenke 1-2)	Estimated blood loss / EBL (mL)	Multifidus FA	-8.789	121.482	-258.986 to 241.407	0.94289800
Group 1 (Lenke 1-2)	Estimated blood loss / EBL (mL)	Paraspinal CSA	7.549	10.545	-14.169 to 29.267	0.48070800
Group 1 (Lenke 1-2)	Estimated blood loss / EBL (mL)	Paraspinal Muscle Index (PPMI)	-4.034	34.327	-74.731 to 66.664	0.90739300
Group 1 (Lenke 1-2)	Estimated blood loss / EBL (mL)	Paraspinal %Fat	-2.368	7.850	-18.536 to 13.799	0.76539500
Group 1 (Lenke 1-2)	Estimated blood loss / EBL (mL)	Paraspinal FA	2.629	30.963	-61.140 to 66.398	0.93301800
Group 1 (Lenke 1-2)	Estimated blood loss / EBL (mL)	Paraspinal %FCSA	2.368	7.850	-13.799 to 18.536	0.76539500
Group 1 (Lenke 1-2)	Estimated blood loss / EBL (mL)	Paraspinal FCSA	10.747	12.801	-15.618 to 37.111	0.40915300
Group 1 (Lenke 1-2)	Estimated blood loss / EBL (mL)	Paraspinal Modified Goutallier Classification	140.027	125.478	-118.400 to 398.453	0.27505800
Group 1 (Lenke 1-2)	Estimated blood loss / EBL (mL)	Quadratus Lumborum CSA	40.018	42.611	-47.741 to 127.778	0.35664000
Group 1 (Lenke 1-2)	Estimated blood loss / EBL (mL)	Quadratus Lumborum %Fat	22.065	14.534	-7.868 to 51.998	0.14151500
Group 1 (Lenke 1-2)	Estimated blood loss / EBL (mL)	Quadratus Lumborum %FCSA	-22.065	14.534	-51.998 to 7.868	0.14151500
Group 1 (Lenke 1-2)	Estimated blood loss / EBL (mL)	Quadratus Lumborum FCSA	40.361	44.461	-51.207 to 131.930	0.37265500
Group 1 (Lenke 1-2)	Estimated blood loss / EBL (mL)	Quadratus Lumborum Modified Goutallier Classification	110.334	157.260	-213.548 to 434.217	0.48940600
Group 1 (Lenke 1-2)	Estimated blood loss / EBL (mL)	Quadratus Lumborum FA	339.674	467.426	-623.007 to 1302.355	0.47416500
Group 1 (Lenke 1-2)	Length of stay / LOS (days)	Psoas CSA	-0.022	0.056	-0.138 to 0.094	0.70276800
Group 1 (Lenke 1-2)	Length of stay / LOS (days)	Psoas Muscle Index (PMI)	-0.183	0.181	-0.555 to 0.189	0.32073900
Group 1 (Lenke 1-2)	Length of stay / LOS (days)	Psoas %Fat	0.053	0.044	-0.037 to 0.143	0.23809400
Group 1	Length of stay / LOS	Psoas FCSA	-0.032	0.058	-0.151 to	0.58837000

Lenke group	Outcome	Predictor	Beta	SE	95% CI	p-value
(Lenke 1-2)	(days)				0.088	
Group 1 (Lenke 1-2)	Length of stay / LOS (days)	Psoas PVR	-0.696	0.679	-2.094 to 0.703	0.31531200
Group 1 (Lenke 1-2)	Length of stay / LOS (days)	Psoas-to-Paraspinal Muscle Ratio (PPMR)	0.003	1.227	-2.524 to 2.531	0.99780300
Group 1 (Lenke 1-2)	Length of stay / LOS (days)	Psoas Modified Goutallier Classification	0.633	0.361	-0.110 to 1.375	0.09172800
Group 1 (Lenke 1-2)	Length of stay / LOS (days)	Psoas FA	0.663	0.492	-0.350 to 1.675	0.18986800
Group 1 (Lenke 1-2)	Length of stay / LOS (days)	Psoas %FCSA	-0.053	0.044	-0.143 to 0.037	0.23809400
Group 1 (Lenke 1-2)	Length of stay / LOS (days)	Erector Spinae CSA	-0.007	0.037	-0.083 to 0.069	0.85211900
Group 1 (Lenke 1-2)	Length of stay / LOS (days)	Erector Spinae Modified Goutallier Classification	0.436	0.403	-0.393 to 1.266	0.28899900
Group 1 (Lenke 1-2)	Length of stay / LOS (days)	Erector Spinae %Fat	-0.010	0.026	-0.063 to 0.044	0.71496900
Group 1 (Lenke 1-2)	Length of stay / LOS (days)	Erector Spinae %FCSA	0.010	0.026	-0.044 to 0.063	0.71496900
Group 1 (Lenke 1-2)	Length of stay / LOS (days)	Erector Spinae FCSA	-0.004	0.042	-0.092 to 0.083	0.92297500
Group 1 (Lenke 1-2)	Length of stay / LOS (days)	Erector Spinae FA	-0.056	0.140	-0.344 to 0.233	0.69446900
Group 1 (Lenke 1-2)	Length of stay / LOS (days)	Multifidus CSA	-0.133	0.149	-0.440 to 0.173	0.37897700
Group 1 (Lenke 1-2)	Length of stay / LOS (days)	Multifidus Modified Goutallier Classification	0.067	0.322	-0.596 to 0.730	0.83618100
Group 1 (Lenke 1-2)	Length of stay / LOS (days)	Multifidus %Fat	0.009	0.019	-0.030 to 0.048	0.64715300
Group 1 (Lenke 1-2)	Length of stay / LOS (days)	Multifidus %FCSA	-0.009	0.019	-0.048 to 0.030	0.64715300
Group 1 (Lenke 1-2)	Length of stay / LOS (days)	Multifidus FCSA	-0.171	0.176	-0.532 to 0.191	0.33964300
Group 1 (Lenke 1-2)	Length of stay / LOS (days)	Multifidus FA	-0.067	0.372	-0.832 to 0.698	0.85813800
Group 1 (Lenke 1-2)	Length of stay / LOS (days)	Paraspinal CSA	-0.012	0.033	-0.079 to 0.055	0.72343100
Group 1 (Lenke 1-2)	Length of stay / LOS (days)	Paraspinal Muscle Index (PPMI)	-0.094	0.103	-0.307 to 0.119	0.36993700
Group 1 (Lenke 1-2)	Length of stay / LOS (days)	Paraspinal %Fat	0.003	0.024	-0.047 to 0.052	0.90626000
Group 1 (Lenke 1-2)	Length of stay / LOS (days)	Paraspinal FA	-0.001	0.095	-0.197 to 0.194	0.98875200
Group 1 (Lenke 1-2)	Length of stay / LOS (days)	Paraspinal %FCSA	-0.003	0.024	-0.052 to 0.047	0.90626000
Group 1 (Lenke 1-2)	Length of stay / LOS (days)	Paraspinal FCSA	-0.017	0.040	-0.099 to 0.064	0.67045200
Group 1 (Lenke 1-2)	Length of stay / LOS (days)	Paraspinal Modified Goutallier Classification	0.254	0.390	-0.549 to 1.058	0.52080200

Lenke group	Outcome	Predictor	Beta	SE	95% CI	p-value
Group 1 (Lenke 1-2)	Length of stay / LOS (days)	Quadratus Lumborum CSA	-0.079	0.132	-0.350 to 0.193	0.55628000
Group 1 (Lenke 1-2)	Length of stay / LOS (days)	Quadratus Lumborum %Fat	0.042	0.046	-0.053 to 0.136	0.37284000
Group 1 (Lenke 1-2)	Length of stay / LOS (days)	Quadratus Lumborum %FCSA	-0.042	0.046	-0.136 to 0.053	0.37284000
Group 1 (Lenke 1-2)	Length of stay / LOS (days)	Quadratus Lumborum FCSA	-0.101	0.137	-0.383 to 0.181	0.46703600
Group 1 (Lenke 1-2)	Length of stay / LOS (days)	Quadratus Lumborum Modified Goutallier Classification	0.596	0.471	-0.375 to 1.566	0.21789300
Group 1 (Lenke 1-2)	Length of stay / LOS (days)	Quadratus Lumborum FA	1.714	1.404	-1.178 to 4.607	0.23354700
Group 1 (Lenke 1-2)	Time to first assisted ambulation (days)	Psoas CSA	-0.008	0.061	-0.133 to 0.117	0.89453000
Group 1 (Lenke 1-2)	Time to first assisted ambulation (days)	Psoas Muscle Index (PMI)	-0.063	0.197	-0.468 to 0.343	0.75294000
Group 1 (Lenke 1-2)	Time to first assisted ambulation (days)	Psoas %Fat	0.071	0.046	-0.025 to 0.166	0.13898100
Group 1 (Lenke 1-2)	Time to first assisted ambulation (days)	Psoas FCSA	-0.016	0.062	-0.144 to 0.113	0.80291700
Group 1 (Lenke 1-2)	Time to first assisted ambulation (days)	Psoas PVR	-0.223	0.741	-1.749 to 1.303	0.76576200
Group 1 (Lenke 1-2)	Time to first assisted ambulation (days)	Psoas-to-Paraspinal Muscle Ratio (PPMR)	-1.029	1.298	-3.702 to 1.645	0.43562600
Group 1 (Lenke 1-2)	Time to first assisted ambulation (days)	Psoas Modified Goutallier Classification	-0.328	0.404	-1.160 to 0.505	0.42507200
Group 1 (Lenke 1-2)	Time to first assisted ambulation (days)	Psoas FA	0.544	0.534	-0.557 to 1.645	0.31842500
Group 1 (Lenke 1-2)	Time to first assisted ambulation (days)	Psoas %FCSA	-0.071	0.046	-0.166 to 0.025	0.13898100
Group 1 (Lenke 1-2)	Time to first assisted ambulation (days)	Erector Spinae CSA	0.009	0.039	-0.072 to 0.090	0.81445000
Group 1 (Lenke 1-2)	Time to first assisted ambulation (days)	Erector Spinae Modified Goutallier Classification	-0.259	0.438	-1.162 to 0.643	0.55931800
Group 1 (Lenke 1-2)	Time to first assisted ambulation (days)	Erector Spinae %Fat	-0.015	0.028	-0.072 to 0.042	0.59779100
Group 1 (Lenke 1-2)	Time to first assisted ambulation (days)	Erector Spinae %FCSA	0.015	0.028	-0.042 to 0.072	0.59779100
Group 1 (Lenke 1-2)	Time to first assisted ambulation (days)	Erector Spinae FCSA	0.018	0.045	-0.075 to 0.112	0.68994000
Group 1 (Lenke 1-2)	Time to first assisted ambulation (days)	Erector Spinae FA	-0.064	0.150	-0.373 to 0.245	0.67290600
Group 1 (Lenke 1-2)	Time to first assisted ambulation (days)	Multifidus CSA	0.182	0.158	-0.143 to 0.507	0.26006200
Group 1 (Lenke 1-2)	Time to first assisted ambulation (days)	Multifidus Modified Goutallier Classification	-0.468	0.332	-1.152 to 0.216	0.17077800
Group 1 (Lenke 1-2)	Time to first assisted ambulation (days)	Multifidus %Fat	-0.023	0.020	-0.064 to 0.018	0.26377400
Group 1	Time to first assisted	Multifidus %FCSA	0.023	0.020	-0.018 to	0.26377400

Lenke group	Outcome	Predictor	Beta	SE	95% CI	p-value
(Lenke 1-2)	ambulation (days)				0.064	
Group 1 (Lenke 1-2)	Time to first assisted ambulation (days)	Multifidus FCSA	0.272	0.184	-0.106 to 0.650	0.15147300
Group 1 (Lenke 1-2)	Time to first assisted ambulation (days)	Multifidus FA	-0.075	0.398	-0.895 to 0.744	0.85108600
Group 1 (Lenke 1-2)	Time to first assisted ambulation (days)	Paraspinal CSA	0.016	0.035	-0.056 to 0.087	0.65371700
Group 1 (Lenke 1-2)	Time to first assisted ambulation (days)	Paraspinal Muscle Index (PPMI)	0.057	0.112	-0.174 to 0.287	0.61769900
Group 1 (Lenke 1-2)	Time to first assisted ambulation (days)	Paraspinal %Fat	-0.024	0.025	-0.077 to 0.028	0.34425600
Group 1 (Lenke 1-2)	Time to first assisted ambulation (days)	Paraspinal FA	-0.060	0.101	-0.268 to 0.147	0.55584200
Group 1 (Lenke 1-2)	Time to first assisted ambulation (days)	Paraspinal %FCSA	0.024	0.025	-0.028 to 0.077	0.34425600
Group 1 (Lenke 1-2)	Time to first assisted ambulation (days)	Paraspinal FCSA	0.034	0.042	-0.052 to 0.121	0.42563700
Group 1 (Lenke 1-2)	Time to first assisted ambulation (days)	Paraspinal Modified Goutallier Classification	-0.475	0.411	-1.320 to 0.371	0.25842900
Group 1 (Lenke 1-2)	Time to first assisted ambulation (days)	Quadratus Lumborum CSA	0.160	0.138	-0.125 to 0.445	0.25769900
Group 1 (Lenke 1-2)	Time to first assisted ambulation (days)	Quadratus Lumborum %Fat	0.054	0.049	-0.046 to 0.154	0.28033600
Group 1 (Lenke 1-2)	Time to first assisted ambulation (days)	Quadratus Lumborum %FCSA	-0.054	0.049	-0.154 to 0.046	0.28033600
Group 1 (Lenke 1-2)	Time to first assisted ambulation (days)	Quadratus Lumborum FCSA	0.149	0.145	-0.150 to 0.447	0.31535900
Group 1 (Lenke 1-2)	Time to first assisted ambulation (days)	Quadratus Lumborum Modified Goutallier Classification	-0.149	0.520	-1.219 to 0.922	0.77716600
Group 1 (Lenke 1-2)	Time to first assisted ambulation (days)	Quadratus Lumborum FA	2.788	1.444	-0.186 to 5.763	0.06496180
Group 2 (Lenke 5)	Surgery duration (min)	Psoas CSA	-1.811	8.571	-20.909 to 17.287	0.83688200
Group 2 (Lenke 5)	Surgery duration (min)	Psoas Muscle Index (PMI)	-18.413	25.103	-74.346 to 37.520	0.48009900
Group 2 (Lenke 5)	Surgery duration (min)	Psoas %Fat	0.659	6.117	-12.971 to 14.290	0.91631500
Group 2 (Lenke 5)	Surgery duration (min)	Psoas FCSA	-1.870	8.499	-20.807 to 17.068	0.83031500
Group 2 (Lenke 5)	Surgery duration (min)	Psoas PVR	60.185	73.925	-104.531 to 224.902	0.43452500
Group 2 (Lenke 5)	Surgery duration (min)	Psoas-to-Paraspinal Muscle Ratio (PPMR)	80.396	91.187	-122.782 to 283.574	0.39865700
Group 2 (Lenke 5)	Surgery duration (min)	Psoas Modified Goutallier Classification	-121.799	47.618	-227.898 to -15.701	0.02847720
Group 2 (Lenke 5)	Surgery duration (min)	Psoas FA	10.358	92.357	-195.425 to 216.141	0.91292300
Group 2 (Lenke 5)	Surgery duration (min)	Psoas %FCSA	-0.659	6.117	-14.290 to 12.971	0.91631500

Lenke group	Outcome	Predictor	Beta	SE	95% CI	p-value
Group 2 (Lenke 5)	Surgery duration (min)	Erector Spinae CSA	-4.364	3.702	-12.613 to 3.884	0.26570800
Group 2 (Lenke 5)	Surgery duration (min)	Erector Spinae Modified Goutallier Classification	1.907	38.423	-83.705 to 87.518	0.96140100
Group 2 (Lenke 5)	Surgery duration (min)	Erector Spinae %Fat	2.172	1.760	-1.749 to 6.094	0.24524800
Group 2 (Lenke 5)	Surgery duration (min)	Erector Spinae %FCSA	-2.172	1.760	-6.094 to 1.749	0.24524800
Group 2 (Lenke 5)	Surgery duration (min)	Erector Spinae FCSA	-4.300	3.419	-11.917 to 3.317	0.23699100
Group 2 (Lenke 5)	Surgery duration (min)	Erector Spinae FA	25.266	24.480	-29.279 to 79.810	0.32633800
Group 2 (Lenke 5)	Surgery duration (min)	Multifidus CSA	-1.176	11.294	-26.340 to 23.987	0.91909200
Group 2 (Lenke 5)	Surgery duration (min)	Multifidus Modified Goutallier Classification	2.774	26.157	-55.507 to 61.055	0.91764100
Group 2 (Lenke 5)	Surgery duration (min)	Multifidus %Fat	1.243	1.390	-1.855 to 4.340	0.39239200
Group 2 (Lenke 5)	Surgery duration (min)	Multifidus %FCSA	-1.243	1.390	-4.340 to 1.855	0.39239200
Group 2 (Lenke 5)	Surgery duration (min)	Multifidus FCSA	-9.663	16.375	-46.149 to 26.824	0.56824300
Group 2 (Lenke 5)	Surgery duration (min)	Multifidus FA	14.697	23.502	-37.669 to 67.064	0.54574100
Group 2 (Lenke 5)	Surgery duration (min)	Paraspinal CSA	-3.138	3.143	-10.142 to 3.866	0.34165200
Group 2 (Lenke 5)	Surgery duration (min)	Paraspinal Muscle Index (PPMI)	-11.105	7.955	-28.830 to 6.620	0.19295800
Group 2 (Lenke 5)	Surgery duration (min)	Paraspinal %Fat	1.884	1.666	-1.827 to 5.595	0.28445100
Group 2 (Lenke 5)	Surgery duration (min)	Paraspinal FA	7.017	13.584	-23.250 to 37.285	0.61668700
Group 2 (Lenke 5)	Surgery duration (min)	Paraspinal %FCSA	-1.884	1.666	-5.595 to 1.827	0.28445100
Group 2 (Lenke 5)	Surgery duration (min)	Paraspinal FCSA	-3.770	3.186	-10.868 to 3.328	0.26400400
Group 2 (Lenke 5)	Surgery duration (min)	Paraspinal Modified Goutallier Classification	-6.990	41.290	-98.989 to 85.010	0.86895100
Group 2 (Lenke 5)	Surgery duration (min)	Quadratus Lumborum CSA	-6.644	5.090	-17.986 to 4.698	0.22104400
Group 2 (Lenke 5)	Surgery duration (min)	Quadratus Lumborum %Fat	2.884	2.218	-2.058 to 7.826	0.22270300
Group 2 (Lenke 5)	Surgery duration (min)	Quadratus Lumborum %FCSA	-2.884	2.218	-7.826 to 2.058	0.22270300
Group 2 (Lenke 5)	Surgery duration (min)	Quadratus Lumborum FCSA	-6.722	5.043	-17.959 to 4.514	0.21210900
Group 2 (Lenke 5)	Surgery duration (min)	Quadratus Lumborum Modified Goutallier Classification	-39.836	41.742	-132.842 to 53.170	0.36240800
Group 2	Surgery duration (min)	Quadratus Lumborum FA	36.543	81.842	-145.812 to	0.66474200

Lenke group	Outcome	Predictor	Beta	SE	95% CI	p-value
(Lenke 5)					218.899	
Group 2 (Lenke 5)	Estimated blood loss / EBL (mL)	Psoas CSA	10.952	19.515	-33.194 to 55.099	0.58835500
Group 2 (Lenke 5)	Estimated blood loss / EBL (mL)	Psoas Muscle Index (PMI)	28.774	60.244	-107.509 to 165.056	0.64431100
Group 2 (Lenke 5)	Estimated blood loss / EBL (mL)	Psoas %Fat	-16.803	12.965	-46.132 to 12.526	0.22722500
Group 2 (Lenke 5)	Estimated blood loss / EBL (mL)	Psoas FCSA	13.573	19.167	-29.787 to 56.932	0.49680300
Group 2 (Lenke 5)	Estimated blood loss / EBL (mL)	Psoas PVR	83.759	179.617	-322.563 to 490.082	0.65206600
Group 2 (Lenke 5)	Estimated blood loss / EBL (mL)	Psoas-to-Paraspinal Muscle Ratio (PPMR)	-567.979	124.560	-849.754 to -286.205	0.00136651
Group 2 (Lenke 5)	Estimated blood loss / EBL (mL)	Psoas Modified Goutallier Classification	207.115	168.021	-172.975 to 587.205	0.24892600
Group 2 (Lenke 5)	Estimated blood loss / EBL (mL)	Psoas FA	-327.825	183.119	-742.070 to 86.419	0.10703200
Group 2 (Lenke 5)	Estimated blood loss / EBL (mL)	Psoas %FCSA	16.803	12.965	-12.526 to 46.132	0.22722500
Group 2 (Lenke 5)	Estimated blood loss / EBL (mL)	Erector Spinae CSA	18.140	7.615	0.914 to 35.366	0.04108230
Group 2 (Lenke 5)	Estimated blood loss / EBL (mL)	Erector Spinae Modified Goutallier Classification	-22.002	88.322	-221.800 to 177.797	0.80887100
Group 2 (Lenke 5)	Estimated blood loss / EBL (mL)	Erector Spinae %Fat	-4.627	4.415	-14.614 to 5.360	0.32196100
Group 2 (Lenke 5)	Estimated blood loss / EBL (mL)	Erector Spinae %FCSA	4.627	4.415	-5.360 to 14.614	0.32196100
Group 2 (Lenke 5)	Estimated blood loss / EBL (mL)	Erector Spinae FCSA	16.103	7.386	-0.606 to 32.812	0.05716380
Group 2 (Lenke 5)	Estimated blood loss / EBL (mL)	Erector Spinae FA	-5.038	62.442	-146.292 to 136.216	0.93746000
Group 2 (Lenke 5)	Estimated blood loss / EBL (mL)	Multifidus CSA	58.351	17.362	19.076 to 97.625	0.00837723
Group 2 (Lenke 5)	Estimated blood loss / EBL (mL)	Multifidus Modified Goutallier Classification	-8.717	60.317	-145.164 to 127.729	0.88826900
Group 2 (Lenke 5)	Estimated blood loss / EBL (mL)	Multifidus %Fat	-3.290	3.284	-10.719 to 4.138	0.34251400
Group 2 (Lenke 5)	Estimated blood loss / EBL (mL)	Multifidus %FCSA	3.290	3.284	-4.138 to 10.719	0.34251400
Group 2 (Lenke 5)	Estimated blood loss / EBL (mL)	Multifidus FCSA	98.063	21.414	49.622 to 146.504	0.00132885
Group 2 (Lenke 5)	Estimated blood loss / EBL (mL)	Multifidus FA	68.586	51.468	-47.843 to 185.014	0.21541900
Group 2 (Lenke 5)	Estimated blood loss / EBL (mL)	Paraspinal CSA	17.646	5.380	5.475 to 29.816	0.00953117
Group 2 (Lenke 5)	Estimated blood loss / EBL (mL)	Paraspinal Muscle Index (PPMI)	48.697	14.730	15.375 to 82.019	0.00914277
Group 2 (Lenke 5)	Estimated blood loss / EBL (mL)	Paraspinal %Fat	-4.561	4.057	-13.738 to 4.616	0.28995500

Lenke group	Outcome	Predictor	Beta	SE	95% CI	p-value
Group 2 (Lenke 5)	Estimated blood loss / EBL (mL)	Paraspinal FA	29.315	30.641	-40.000 to 98.630	0.36370300
Group 2 (Lenke 5)	Estimated blood loss / EBL (mL)	Paraspinal %FCSA	4.561	4.057	-4.616 to 13.738	0.28995500
Group 2 (Lenke 5)	Estimated blood loss / EBL (mL)	Paraspinal FCSA	17.491	6.012	3.890 to 31.092	0.01733730
Group 2 (Lenke 5)	Estimated blood loss / EBL (mL)	Paraspinal Modified Goutallier Classification	-2.475	95.485	-218.477 to 213.528	0.97988900
Group 2 (Lenke 5)	Estimated blood loss / EBL (mL)	Quadratus Lumborum CSA	29.432	9.617	7.677 to 51.188	0.01356220
Group 2 (Lenke 5)	Estimated blood loss / EBL (mL)	Quadratus Lumborum %Fat	-5.299	5.713	-18.222 to 7.625	0.37788900
Group 2 (Lenke 5)	Estimated blood loss / EBL (mL)	Quadratus Lumborum %FCSA	5.299	5.713	-7.625 to 18.222	0.37788900
Group 2 (Lenke 5)	Estimated blood loss / EBL (mL)	Quadratus Lumborum FCSA	29.302	9.601	7.582 to 51.021	0.01375030
Group 2 (Lenke 5)	Estimated blood loss / EBL (mL)	Quadratus Lumborum Modified Goutallier Classification	-152.840	91.840	-360.597 to 54.917	0.13043100
Group 2 (Lenke 5)	Estimated blood loss / EBL (mL)	Quadratus Lumborum FA	-9.950	192.465	-445.336 to 425.436	0.95989700
Group 2 (Lenke 5)	Length of stay / LOS (days)	Psoas CSA	-0.175	0.144	-0.500 to 0.150	0.25319700
Group 2 (Lenke 5)	Length of stay / LOS (days)	Psoas Muscle Index (PMI)	-0.323	0.464	-1.374 to 0.727	0.50396300
Group 2 (Lenke 5)	Length of stay / LOS (days)	Psoas %Fat	-0.031	0.110	-0.279 to 0.218	0.78652000
Group 2 (Lenke 5)	Length of stay / LOS (days)	Psoas FCSA	-0.165	0.144	-0.490 to 0.161	0.28149900
Group 2 (Lenke 5)	Length of stay / LOS (days)	Psoas PVR	-1.010	1.380	-4.131 to 2.112	0.48291700
Group 2 (Lenke 5)	Length of stay / LOS (days)	Psoas-to-Paraspinal Muscle Ratio (PPMR)	-2.560	1.552	-6.070 to 0.951	0.13342800
Group 2 (Lenke 5)	Length of stay / LOS (days)	Psoas Modified Goutallier Classification	1.209	1.361	-1.869 to 4.288	0.39731100
Group 2 (Lenke 5)	Length of stay / LOS (days)	Psoas FA	-0.927	1.637	-4.631 to 2.777	0.58513800
Group 2 (Lenke 5)	Length of stay / LOS (days)	Psoas %FCSA	0.031	0.110	-0.218 to 0.279	0.78652000
Group 2 (Lenke 5)	Length of stay / LOS (days)	Erector Spinae CSA	0.017	0.076	-0.154 to 0.188	0.82719900
Group 2 (Lenke 5)	Length of stay / LOS (days)	Erector Spinae Modified Goutallier Classification	-0.730	0.648	-2.197 to 0.736	0.28897400
Group 2 (Lenke 5)	Length of stay / LOS (days)	Erector Spinae %Fat	-0.013	0.036	-0.095 to 0.069	0.73306300
Group 2 (Lenke 5)	Length of stay / LOS (days)	Erector Spinae %FCSA	0.013	0.036	-0.069 to 0.095	0.73306300
Group 2 (Lenke 5)	Length of stay / LOS (days)	Erector Spinae FCSA	0.016	0.071	-0.145 to 0.177	0.82836300
Group 2	Length of stay / LOS	Erector Spinae FA	-0.041	0.488	-1.144 to	0.93564000

Lenke group	Outcome	Predictor	Beta	SE	95% CI	p-value
(Lenke 5)	(days)				1.063	
Group 2 (Lenke 5)	Length of stay / LOS (days)	Multifidus CSA	0.137	0.199	-0.312 to 0.586	0.50696100
Group 2 (Lenke 5)	Length of stay / LOS (days)	Multifidus Modified Goutallier Classification	-0.279	0.463	-1.326 to 0.767	0.56067700
Group 2 (Lenke 5)	Length of stay / LOS (days)	Multifidus %Fat	-0.010	0.027	-0.071 to 0.050	0.70595600
Group 2 (Lenke 5)	Length of stay / LOS (days)	Multifidus %FCSA	0.010	0.027	-0.050 to 0.071	0.70595600
Group 2 (Lenke 5)	Length of stay / LOS (days)	Multifidus FCSA	0.250	0.294	-0.415 to 0.914	0.41721600
Group 2 (Lenke 5)	Length of stay / LOS (days)	Multifidus FA	0.121	0.438	-0.870 to 1.113	0.78814500
Group 2 (Lenke 5)	Length of stay / LOS (days)	Paraspinal CSA	0.024	0.062	-0.115 to 0.164	0.70349900
Group 2 (Lenke 5)	Length of stay / LOS (days)	Paraspinal Muscle Index (PPMI)	0.145	0.164	-0.226 to 0.517	0.39950700
Group 2 (Lenke 5)	Length of stay / LOS (days)	Paraspinal %Fat	-0.014	0.034	-0.090 to 0.062	0.69337300
Group 2 (Lenke 5)	Length of stay / LOS (days)	Paraspinal FA	0.020	0.251	-0.548 to 0.588	0.93799600
Group 2 (Lenke 5)	Length of stay / LOS (days)	Paraspinal %FCSA	0.014	0.034	-0.062 to 0.090	0.69337300
Group 2 (Lenke 5)	Length of stay / LOS (days)	Paraspinal FCSA	0.025	0.065	-0.121 to 0.172	0.70429400
Group 2 (Lenke 5)	Length of stay / LOS (days)	Paraspinal Modified Goutallier Classification	-0.438	0.732	-2.093 to 1.217	0.56408800
Group 2 (Lenke 5)	Length of stay / LOS (days)	Quadratus Lumborum CSA	-0.002	0.107	-0.245 to 0.241	0.98719100
Group 2 (Lenke 5)	Length of stay / LOS (days)	Quadratus Lumborum %Fat	0.016	0.046	-0.089 to 0.121	0.73601100
Group 2 (Lenke 5)	Length of stay / LOS (days)	Quadratus Lumborum %FCSA	-0.016	0.046	-0.121 to 0.089	0.73601100
Group 2 (Lenke 5)	Length of stay / LOS (days)	Quadratus Lumborum FCSA	-0.005	0.107	-0.247 to 0.237	0.96517500
Group 2 (Lenke 5)	Length of stay / LOS (days)	Quadratus Lumborum Modified Goutallier Classification	0.651	0.791	-1.139 to 2.441	0.43197300
Group 2 (Lenke 5)	Length of stay / LOS (days)	Quadratus Lumborum FA	0.601	1.491	-2.771 to 3.973	0.69622800
Group 2 (Lenke 5)	Time to first assisted ambulation (days)	Psoas CSA	-0.048	0.142	-0.371 to 0.274	0.74183600
Group 2 (Lenke 5)	Time to first assisted ambulation (days)	Psoas Muscle Index (PMI)	-0.329	0.426	-1.294 to 0.636	0.46003900
Group 2 (Lenke 5)	Time to first assisted ambulation (days)	Psoas %Fat	0.236	0.065	0.090 to 0.383	0.00530508
Group 2 (Lenke 5)	Time to first assisted ambulation (days)	Psoas FCSA	-0.072	0.140	-0.389 to 0.245	0.61915200
Group 2 (Lenke 5)	Time to first assisted ambulation (days)	Psoas PVR	0.172	1.311	-2.794 to 3.137	0.89860500

Lenke group	Outcome	Predictor	Beta	SE	95% CI	p-value
Group 2 (Lenke 5)	Time to first assisted ambulation (days)	Psoas-to-Paraspinal Muscle Ratio (PPMR)	1.311	1.576	-2.255 to 4.877	0.42714100
Group 2 (Lenke 5)	Time to first assisted ambulation (days)	Psoas Modified Goutallier Classification	0.625	1.295	-2.303 to 3.554	0.64062900
Group 2 (Lenke 5)	Time to first assisted ambulation (days)	Psoas FA	2.874	1.205	0.148 to 5.599	0.04087850
Group 2 (Lenke 5)	Time to first assisted ambulation (days)	Psoas %FCSA	-0.236	0.065	-0.383 to -0.090	0.00530508
Group 2 (Lenke 5)	Time to first assisted ambulation (days)	Erector Spinae CSA	-0.087	0.064	-0.232 to 0.057	0.20372900
Group 2 (Lenke 5)	Time to first assisted ambulation (days)	Erector Spinae Modified Goutallier Classification	0.185	0.637	-1.255 to 1.625	0.77793700
Group 2 (Lenke 5)	Time to first assisted ambulation (days)	Erector Spinae %Fat	0.059	0.028	-0.004 to 0.121	0.06177530
Group 2 (Lenke 5)	Time to first assisted ambulation (days)	Erector Spinae %FCSA	-0.059	0.028	-0.121 to 0.004	0.06177530
Group 2 (Lenke 5)	Time to first assisted ambulation (days)	Erector Spinae FCSA	-0.087	0.059	-0.221 to 0.046	0.17358100
Group 2 (Lenke 5)	Time to first assisted ambulation (days)	Erector Spinae FA	0.478	0.422	-0.476 to 1.432	0.28603200
Group 2 (Lenke 5)	Time to first assisted ambulation (days)	Multifidus CSA	-0.147	0.182	-0.558 to 0.264	0.44011300
Group 2 (Lenke 5)	Time to first assisted ambulation (days)	Multifidus Modified Goutallier Classification	0.148	0.433	-0.831 to 1.128	0.73963500
Group 2 (Lenke 5)	Time to first assisted ambulation (days)	Multifidus %Fat	0.017	0.024	-0.037 to 0.072	0.49005100
Group 2 (Lenke 5)	Time to first assisted ambulation (days)	Multifidus %FCSA	-0.017	0.024	-0.072 to 0.037	0.49005100
Group 2 (Lenke 5)	Time to first assisted ambulation (days)	Multifidus FCSA	-0.321	0.261	-0.911 to 0.269	0.24978600
Group 2 (Lenke 5)	Time to first assisted ambulation (days)	Multifidus FA	-0.018	0.406	-0.937 to 0.902	0.96572200
Group 2 (Lenke 5)	Time to first assisted ambulation (days)	Paraspinal CSA	-0.073	0.052	-0.191 to 0.046	0.19825300
Group 2 (Lenke 5)	Time to first assisted ambulation (days)	Paraspinal Muscle Index (PPMI)	-0.242	0.136	-0.550 to 0.065	0.10859700
Group 2 (Lenke 5)	Time to first assisted ambulation (days)	Paraspinal %Fat	0.039	0.028	-0.026 to 0.103	0.20526000
Group 2 (Lenke 5)	Time to first assisted ambulation (days)	Paraspinal FA	0.053	0.231	-0.471 to 0.576	0.82558100
Group 2 (Lenke 5)	Time to first assisted ambulation (days)	Paraspinal %FCSA	-0.039	0.028	-0.103 to 0.026	0.20526000
Group 2 (Lenke 5)	Time to first assisted ambulation (days)	Paraspinal FCSA	-0.084	0.054	-0.205 to 0.038	0.15348900
Group 2 (Lenke 5)	Time to first assisted ambulation (days)	Paraspinal Modified Goutallier Classification	0.111	0.688	-1.446 to 1.668	0.87550700
Group 2 (Lenke 5)	Time to first assisted ambulation (days)	Quadratus Lumborum CSA	0.033	0.099	-0.190 to 0.256	0.74631400
Group 2	Time to first assisted	Quadratus Lumborum %Fat	0.074	0.035	-0.006 to	0.06486020

Lenke group	Outcome	Predictor	Beta	SE	95% CI	p-value
(Lenke 5)	ambulation (days)				0.154	
Group 2 (Lenke 5)	Time to first assisted ambulation (days)	Quadratus Lumborum %FCSA	-0.074	0.035	-0.154 to 0.006	0.06486020
Group 2 (Lenke 5)	Time to first assisted ambulation (days)	Quadratus Lumborum FCSA	0.024	0.099	-0.199 to 0.247	0.81514800
Group 2 (Lenke 5)	Time to first assisted ambulation (days)	Quadratus Lumborum Modified Goutallier Classification	0.959	0.687	-0.596 to 2.514	0.19635200
Group 2 (Lenke 5)	Time to first assisted ambulation (days)	Quadratus Lumborum FA	1.769	1.258	-1.077 to 4.615	0.19335200
Group 3 (Lenke 3-4-6)	Surgery duration (min)	Psoas CSA	1.495	2.762	-4.124 to 7.114	0.59190000
Group 3 (Lenke 3-4-6)	Surgery duration (min)	Psoas Muscle Index (PMI)	6.840	7.428	-8.273 to 21.953	0.36386700
Group 3 (Lenke 3-4-6)	Surgery duration (min)	Psoas %Fat	-4.845	2.411	-9.750 to 0.061	0.05273070
Group 3 (Lenke 3-4-6)	Surgery duration (min)	Psoas FCSA	2.106	2.779	-3.549 to 7.760	0.45404300
Group 3 (Lenke 3-4-6)	Surgery duration (min)	Psoas PVR	-24.010	32.356	-89.838 to 41.819	0.46330300
Group 3 (Lenke 3-4-6)	Surgery duration (min)	Psoas-to-Paraspinal Muscle Ratio (PPMR)	-10.781	42.374	-96.991 to 75.429	0.80075000
Group 3 (Lenke 3-4-6)	Surgery duration (min)	Psoas Modified Goutallier Classification	-6.078	21.544	-49.910 to 37.754	0.77961500
Group 3 (Lenke 3-4-6)	Surgery duration (min)	Psoas FA	-61.736	26.885	-116.434 to -7.039	0.02814320
Group 3 (Lenke 3-4-6)	Surgery duration (min)	Psoas %FCSA	4.845	2.411	-0.061 to 9.750	0.05273070
Group 3 (Lenke 3-4-6)	Surgery duration (min)	Erector Spinae CSA	-0.252	2.744	-5.836 to 5.332	0.92741900
Group 3 (Lenke 3-4-6)	Surgery duration (min)	Erector Spinae Modified Goutallier Classification	13.347	11.642	-10.339 to 37.032	0.25985800
Group 3 (Lenke 3-4-6)	Surgery duration (min)	Erector Spinae %Fat	1.389	0.764	-0.167 to 2.944	0.07838080
Group 3 (Lenke 3-4-6)	Surgery duration (min)	Erector Spinae %FCSA	-1.389	0.764	-2.944 to 0.167	0.07838080
Group 3 (Lenke 3-4-6)	Surgery duration (min)	Erector Spinae FCSA	-3.822	2.991	-9.906 to 2.263	0.21022300
Group 3 (Lenke 3-4-6)	Surgery duration (min)	Erector Spinae FA	9.799	4.828	-0.024 to 19.622	0.05053930
Group 3 (Lenke 3-4-	Surgery duration (min)	Multifidus CSA	8.465	4.299	-0.282 to	0.05739600

Lenke group	Outcome	Predictor	Beta	SE	95% CI	p-value
6)					17.211	
Group 3 (Lenke 3-4-6)	Surgery duration (min)	Multifidus Modified Goutallier Classification	5.311	12.264	-19.641 to 30.263	0.66780500
Group 3 (Lenke 3-4-6)	Surgery duration (min)	Multifidus %Fat	1.025	0.603	-0.202 to 2.253	0.09847820
Group 3 (Lenke 3-4-6)	Surgery duration (min)	Multifidus %FCSA	-1.025	0.603	-2.253 to 0.202	0.09847820
Group 3 (Lenke 3-4-6)	Surgery duration (min)	Multifidus FCSA	7.723	6.678	-5.862 to 21.309	0.25573200
Group 3 (Lenke 3-4-6)	Surgery duration (min)	Multifidus FA	23.804	8.979	5.536 to 42.073	0.01223050
Group 3 (Lenke 3-4-6)	Surgery duration (min)	Paraspinal CSA	1.621	2.056	-2.562 to 5.803	0.43610400
Group 3 (Lenke 3-4-6)	Surgery duration (min)	Paraspinal Muscle Index (PPMI)	8.877	5.895	-3.116 to 20.871	0.14161000
Group 3 (Lenke 3-4-6)	Surgery duration (min)	Paraspinal %Fat	1.443	0.738	-0.058 to 2.943	0.05899360
Group 3 (Lenke 3-4-6)	Surgery duration (min)	Paraspinal FA	8.415	3.280	1.742 to 15.088	0.01502300
Group 3 (Lenke 3-4-6)	Surgery duration (min)	Paraspinal %FCSA	-1.443	0.738	-2.943 to 0.058	0.05899360
Group 3 (Lenke 3-4-6)	Surgery duration (min)	Paraspinal FCSA	-1.829	2.556	-7.029 to 3.370	0.47919300
Group 3 (Lenke 3-4-6)	Surgery duration (min)	Paraspinal Modified Goutallier Classification	8.769	13.269	-18.226 to 35.764	0.51326700
Group 3 (Lenke 3-4-6)	Surgery duration (min)	Quadratus Lumborum CSA	-1.284	5.275	-12.015 to 9.447	0.80917400
Group 3 (Lenke 3-4-6)	Surgery duration (min)	Quadratus Lumborum %Fat	-0.788	1.509	-3.858 to 2.281	0.60481700
Group 3 (Lenke 3-4-6)	Surgery duration (min)	Quadratus Lumborum %FCSA	0.788	1.509	-2.281 to 3.858	0.60481700
Group 3 (Lenke 3-4-6)	Surgery duration (min)	Quadratus Lumborum FCSA	-0.918	5.756	-12.629 to 10.794	0.87432500
Group 3 (Lenke 3-4-6)	Surgery duration (min)	Quadratus Lumborum Modified Goutallier Classification	-0.953	21.007	-43.693 to 41.786	0.96407500
Group 3 (Lenke 3-4-6)	Surgery duration (min)	Quadratus Lumborum FA	-23.735	35.677	-96.320 to 48.851	0.51050700
Group 3 (Lenke 3-4-	Estimated blood loss / EBL (mL)	Psoas CSA	27.925	16.088	-4.846 to 60.696	0.09223490

Lenke group	Outcome	Predictor	Beta	SE	95% CI	p-value
6)						
Group 3 (Lenke 3-4-6)	Estimated blood loss / EBL (mL)	Psoas Muscle Index (PMI)	89.077	43.239	1.001 to 177.152	0.04760060
Group 3 (Lenke 3-4-6)	Estimated blood loss / EBL (mL)	Psoas %Fat	-19.176	15.988	-51.742 to 13.391	0.23918800
Group 3 (Lenke 3-4-6)	Estimated blood loss / EBL (mL)	Psoas FCSA	29.426	16.267	-3.709 to 62.561	0.07986660
Group 3 (Lenke 3-4-6)	Estimated blood loss / EBL (mL)	Psoas PVR	147.910	196.951	-253.267 to 549.087	0.45814200
Group 3 (Lenke 3-4-6)	Estimated blood loss / EBL (mL)	Psoas-to-Paraspinal Muscle Ratio (PPMR)	164.890	254.764	-354.047 to 683.826	0.52210200
Group 3 (Lenke 3-4-6)	Estimated blood loss / EBL (mL)	Psoas Modified Goutallier Classification	-87.969	129.504	-351.761 to 175.823	0.50184700
Group 3 (Lenke 3-4-6)	Estimated blood loss / EBL (mL)	Psoas FA	-80.940	187.778	-463.432 to 301.551	0.66932800
Group 3 (Lenke 3-4-6)	Estimated blood loss / EBL (mL)	Psoas %FCSA	19.176	15.988	-13.391 to 51.742	0.23918800
Group 3 (Lenke 3-4-6)	Estimated blood loss / EBL (mL)	Erector Spinae CSA	18.369	16.259	-14.749 to 51.488	0.26696800
Group 3 (Lenke 3-4-6)	Estimated blood loss / EBL (mL)	Erector Spinae Modified Goutallier Classification	-103.764	70.775	-247.929 to 40.400	0.15237500
Group 3 (Lenke 3-4-6)	Estimated blood loss / EBL (mL)	Erector Spinae %Fat	-3.129	5.049	-13.412 to 7.155	0.53983200
Group 3 (Lenke 3-4-6)	Estimated blood loss / EBL (mL)	Erector Spinae %FCSA	3.129	5.049	-7.155 to 13.412	0.53983200
Group 3 (Lenke 3-4-6)	Estimated blood loss / EBL (mL)	Erector Spinae FCSA	22.293	18.543	-15.477 to 60.063	0.23808600
Group 3 (Lenke 3-4-6)	Estimated blood loss / EBL (mL)	Erector Spinae FA	5.149	32.789	-61.639 to 71.937	0.87620100
Group 3 (Lenke 3-4-6)	Estimated blood loss / EBL (mL)	Multifidus CSA	20.526	28.787	-38.112 to 79.164	0.48100200
Group 3 (Lenke 3-4-6)	Estimated blood loss / EBL (mL)	Multifidus Modified Goutallier Classification	-110.207	71.900	-256.663 to 36.248	0.13515700
Group 3 (Lenke 3-4-6)	Estimated blood loss / EBL (mL)	Multifidus %Fat	-1.950	3.947	-9.989 to 6.089	0.62463500
Group 3 (Lenke 3-4-6)	Estimated blood loss / EBL (mL)	Multifidus %FCSA	1.950	3.947	-6.089 to 9.989	0.62463500
Group 3 (Lenke 3-4-	Estimated blood loss / EBL (mL)	Multifidus FCSA	34.289	41.525	-50.296 to 118.873	0.41507300

Lenke group	Outcome	Predictor	Beta	SE	95% CI	p-value
6)						
Group 3 (Lenke 3-4-6)	Estimated blood loss / EBL (mL)	Multifidus FA	21.252	65.674	-112.523 to 155.026	0.74835100
Group 3 (Lenke 3-4-6)	Estimated blood loss / EBL (mL)	Paraspinal CSA	14.593	12.383	-10.632 to 39.817	0.24732300
Group 3 (Lenke 3-4-6)	Estimated blood loss / EBL (mL)	Paraspinal Muscle Index (PPMI)	61.463	36.467	-12.818 to 135.744	0.10163300
Group 3 (Lenke 3-4-6)	Estimated blood loss / EBL (mL)	Paraspinal %Fat	-3.034	4.942	-13.101 to 7.033	0.54363200
Group 3 (Lenke 3-4-6)	Estimated blood loss / EBL (mL)	Paraspinal FA	8.584	23.712	-39.715 to 56.883	0.71971000
Group 3 (Lenke 3-4-6)	Estimated blood loss / EBL (mL)	Paraspinal %FCSA	3.034	4.942	-7.033 to 13.101	0.54363200
Group 3 (Lenke 3-4-6)	Estimated blood loss / EBL (mL)	Paraspinal FCSA	18.672	15.323	-12.540 to 49.884	0.23192800
Group 3 (Lenke 3-4-6)	Estimated blood loss / EBL (mL)	Paraspinal Modified Goutallier Classification	-129.745	77.892	-288.407 to 28.916	0.10553400
Group 3 (Lenke 3-4-6)	Estimated blood loss / EBL (mL)	Quadratus Lumborum CSA	29.261	31.492	-34.885 to 93.408	0.35975400
Group 3 (Lenke 3-4-6)	Estimated blood loss / EBL (mL)	Quadratus Lumborum %Fat	-2.111	9.180	-20.811 to 16.588	0.81956300
Group 3 (Lenke 3-4-6)	Estimated blood loss / EBL (mL)	Quadratus Lumborum %FCSA	2.111	9.180	-16.588 to 20.811	0.81956300
Group 3 (Lenke 3-4-6)	Estimated blood loss / EBL (mL)	Quadratus Lumborum FCSA	33.204	34.294	-36.650 to 103.058	0.34018900
Group 3 (Lenke 3-4-6)	Estimated blood loss / EBL (mL)	Quadratus Lumborum Modified Goutallier Classification	-132.570	124.703	-386.581 to 121.441	0.29570000
Group 3 (Lenke 3-4-6)	Estimated blood loss / EBL (mL)	Quadratus Lumborum FA	62.000	218.093	-382.241 to 506.241	0.77802700
Group 3 (Lenke 3-4-6)	Length of stay / LOS (days)	Psoas CSA	0.058	0.079	-0.103 to 0.220	0.46758600
Group 3 (Lenke 3-4-6)	Length of stay / LOS (days)	Psoas Muscle Index (PMI)	0.193	0.216	-0.247 to 0.632	0.37876400
Group 3 (Lenke 3-4-6)	Length of stay / LOS (days)	Psoas %Fat	-0.150	0.073	-0.298 to -0.001	0.04843580
Group 3 (Lenke 3-4-6)	Length of stay / LOS (days)	Psoas FCSA	0.071	0.080	-0.092 to 0.234	0.38065900
Group 3 (Lenke 3-4-	Length of stay / LOS (days)	Psoas PVR	0.628	0.937	-1.280 to 2.536	0.50751500

Lenke group	Outcome	Predictor	Beta	SE	95% CI	p-value
6)						
Group 3 (Lenke 3-4-6)	Length of stay / LOS (days)	Psoas-to-Paraspinal Muscle Ratio (PPMR)	0.296	1.217	-2.182 to 2.774	0.80943900
Group 3 (Lenke 3-4-6)	Length of stay / LOS (days)	Psoas Modified Goutallier Classification	0.528	0.612	-0.720 to 1.775	0.39515600
Group 3 (Lenke 3-4-6)	Length of stay / LOS (days)	Psoas FA	-1.364	0.861	-3.118 to 0.390	0.12307800
Group 3 (Lenke 3-4-6)	Length of stay / LOS (days)	Psoas %FCSA	0.150	0.073	0.001 to 0.298	0.04843580
Group 3 (Lenke 3-4-6)	Length of stay / LOS (days)	Erector Spinae CSA	0.090	0.077	-0.067 to 0.247	0.25286200
Group 3 (Lenke 3-4-6)	Length of stay / LOS (days)	Erector Spinae Modified Goutallier Classification	-0.386	0.340	-1.079 to 0.308	0.26544700
Group 3 (Lenke 3-4-6)	Length of stay / LOS (days)	Erector Spinae %Fat	-0.018	0.024	-0.067 to 0.031	0.45869500
Group 3 (Lenke 3-4-6)	Length of stay / LOS (days)	Erector Spinae %FCSA	0.018	0.024	-0.031 to 0.067	0.45869500
Group 3 (Lenke 3-4-6)	Length of stay / LOS (days)	Erector Spinae FCSA	0.126	0.087	-0.051 to 0.304	0.15733300
Group 3 (Lenke 3-4-6)	Length of stay / LOS (days)	Erector Spinae FA	-0.027	0.156	-0.344 to 0.291	0.86526100
Group 3 (Lenke 3-4-6)	Length of stay / LOS (days)	Multifidus CSA	-0.133	0.136	-0.409 to 0.144	0.33504400
Group 3 (Lenke 3-4-6)	Length of stay / LOS (days)	Multifidus Modified Goutallier Classification	-0.218	0.352	-0.934 to 0.498	0.53950100
Group 3 (Lenke 3-4-6)	Length of stay / LOS (days)	Multifidus %Fat	-0.007	0.019	-0.045 to 0.032	0.73107200
Group 3 (Lenke 3-4-6)	Length of stay / LOS (days)	Multifidus %FCSA	0.007	0.019	-0.032 to 0.045	0.73107200
Group 3 (Lenke 3-4-6)	Length of stay / LOS (days)	Multifidus FCSA	-0.142	0.198	-0.545 to 0.260	0.47651700
Group 3 (Lenke 3-4-6)	Length of stay / LOS (days)	Multifidus FA	-0.333	0.307	-0.958 to 0.292	0.28568400
Group 3 (Lenke 3-4-6)	Length of stay / LOS (days)	Paraspinal CSA	0.027	0.060	-0.095 to 0.149	0.65496000
Group 3 (Lenke 3-4-6)	Length of stay / LOS (days)	Paraspinal Muscle Index (PPMI)	0.114	0.180	-0.252 to 0.480	0.52932200
Group 3 (Lenke 3-4-	Length of stay / LOS (days)	Paraspinal %Fat	-0.014	0.023	-0.062 to 0.034	0.56323300

Lenke group	Outcome	Predictor	Beta	SE	95% CI	p-value
6)						
Group 3 (Lenke 3-4-6)	Length of stay / LOS (days)	Paraspinal FA	-0.037	0.113	-0.266 to 0.193	0.74541600
Group 3 (Lenke 3-4-6)	Length of stay / LOS (days)	Paraspinal %FCSA	0.014	0.023	-0.034 to 0.062	0.56323300
Group 3 (Lenke 3-4-6)	Length of stay / LOS (days)	Paraspinal FCSA	0.058	0.074	-0.093 to 0.208	0.44098600
Group 3 (Lenke 3-4-6)	Length of stay / LOS (days)	Paraspinal Modified Goutallier Classification	-0.223	0.384	-1.005 to 0.558	0.56462200
Group 3 (Lenke 3-4-6)	Length of stay / LOS (days)	Quadratus Lumborum CSA	0.114	0.150	-0.192 to 0.420	0.45237300
Group 3 (Lenke 3-4-6)	Length of stay / LOS (days)	Quadratus Lumborum %Fat	-0.036	0.043	-0.124 to 0.052	0.41327000
Group 3 (Lenke 3-4-6)	Length of stay / LOS (days)	Quadratus Lumborum %FCSA	0.036	0.043	-0.052 to 0.124	0.41327000
Group 3 (Lenke 3-4-6)	Length of stay / LOS (days)	Quadratus Lumborum FCSA	0.149	0.163	-0.183 to 0.481	0.36707600
Group 3 (Lenke 3-4-6)	Length of stay / LOS (days)	Quadratus Lumborum Modified Goutallier Classification	-0.422	0.598	-1.639 to 0.796	0.48587300
Group 3 (Lenke 3-4-6)	Length of stay / LOS (days)	Quadratus Lumborum FA	-0.529	1.033	-2.632 to 1.575	0.61231400
Group 3 (Lenke 3-4-6)	Time to first assisted ambulation (days)	Psoas CSA	0.003	0.041	-0.080 to 0.086	0.94568500
Group 3 (Lenke 3-4-6)	Time to first assisted ambulation (days)	Psoas Muscle Index (PMI)	0.018	0.112	-0.210 to 0.246	0.87433900
Group 3 (Lenke 3-4-6)	Time to first assisted ambulation (days)	Psoas %Fat	-0.048	0.039	-0.127 to 0.031	0.22038300
Group 3 (Lenke 3-4-6)	Time to first assisted ambulation (days)	Psoas FCSA	0.006	0.041	-0.079 to 0.090	0.89220100
Group 3 (Lenke 3-4-6)	Time to first assisted ambulation (days)	Psoas PVR	-0.021	0.483	-1.005 to 0.962	0.96540600
Group 3 (Lenke 3-4-6)	Time to first assisted ambulation (days)	Psoas-to-Paraspinal Muscle Ratio (PPMR)	-0.325	0.621	-1.589 to 0.939	0.60372200
Group 3 (Lenke 3-4-6)	Time to first assisted ambulation (days)	Psoas Modified Goutallier Classification	-0.026	0.317	-0.672 to 0.620	0.93517700
Group 3 (Lenke 3-4-6)	Time to first assisted ambulation (days)	Psoas FA	-0.338	0.454	-1.262 to 0.587	0.46238300
Group 3 (Lenke 3-4-	Time to first assisted ambulation (days)	Psoas %FCSA	0.048	0.039	-0.031 to 0.127	0.22038300

Lenke group	Outcome	Predictor	Beta	SE	95% CI	p-value
6)						
Group 3 (Lenke 3-4-6)	Time to first assisted ambulation (days)	Erector Spinae CSA	0.018	0.040	-0.064 to 0.099	0.66571900
Group 3 (Lenke 3-4-6)	Time to first assisted ambulation (days)	Erector Spinae Modified Goutallier Classification	-0.049	0.177	-0.411 to 0.312	0.78317700
Group 3 (Lenke 3-4-6)	Time to first assisted ambulation (days)	Erector Spinae %Fat	-0.007	0.012	-0.032 to 0.018	0.55117800
Group 3 (Lenke 3-4-6)	Time to first assisted ambulation (days)	Erector Spinae %FCSA	0.007	0.012	-0.018 to 0.032	0.55117800
Group 3 (Lenke 3-4-6)	Time to first assisted ambulation (days)	Erector Spinae FCSA	0.025	0.046	-0.068 to 0.119	0.58472300
Group 3 (Lenke 3-4-6)	Time to first assisted ambulation (days)	Erector Spinae FA	-0.007	0.080	-0.170 to 0.155	0.92833800
Group 3 (Lenke 3-4-6)	Time to first assisted ambulation (days)	Multifidus CSA	0.073	0.069	-0.068 to 0.215	0.29737600
Group 3 (Lenke 3-4-6)	Time to first assisted ambulation (days)	Multifidus Modified Goutallier Classification	0.013	0.181	-0.356 to 0.381	0.94503500
Group 3 (Lenke 3-4-6)	Time to first assisted ambulation (days)	Multifidus %Fat	0.001	0.010	-0.019 to 0.021	0.91519500
Group 3 (Lenke 3-4-6)	Time to first assisted ambulation (days)	Multifidus %FCSA	-0.001	0.010	-0.021 to 0.019	0.91519500
Group 3 (Lenke 3-4-6)	Time to first assisted ambulation (days)	Multifidus FCSA	0.080	0.101	-0.126 to 0.286	0.43289700
Group 3 (Lenke 3-4-6)	Time to first assisted ambulation (days)	Multifidus FA	0.180	0.157	-0.139 to 0.499	0.25832700
Group 3 (Lenke 3-4-6)	Time to first assisted ambulation (days)	Paraspinal CSA	0.024	0.030	-0.038 to 0.086	0.43343400
Group 3 (Lenke 3-4-6)	Time to first assisted ambulation (days)	Paraspinal Muscle Index (PPMI)	0.085	0.091	-0.101 to 0.271	0.35801500
Group 3 (Lenke 3-4-6)	Time to first assisted ambulation (days)	Paraspinal %Fat	-0.003	0.012	-0.027 to 0.022	0.82264300
Group 3 (Lenke 3-4-6)	Time to first assisted ambulation (days)	Paraspinal FA	0.027	0.058	-0.090 to 0.145	0.63593900
Group 3 (Lenke 3-4-6)	Time to first assisted ambulation (days)	Paraspinal %FCSA	0.003	0.012	-0.022 to 0.027	0.82264300
Group 3 (Lenke 3-4-6)	Time to first assisted ambulation (days)	Paraspinal FCSA	0.025	0.038	-0.052 to 0.102	0.51152600
Group 3 (Lenke 3-4-	Time to first assisted ambulation (days)	Paraspinal Modified Goutallier Classification	0.017	0.197	-0.385 to 0.419	0.93195000

Lenke group	Outcome	Predictor	Beta	SE	95% CI	p-value
6)						
Group 3 (Lenke 3-4-6)	Time to first assisted ambulation (days)	Quadratus Lumborum CSA	0.145	0.073	-0.004 to 0.294	0.05619400
Group 3 (Lenke 3-4-6)	Time to first assisted ambulation (days)	Quadratus Lumborum %Fat	-0.008	0.022	-0.053 to 0.038	0.73318600
Group 3 (Lenke 3-4-6)	Time to first assisted ambulation (days)	Quadratus Lumborum %FCSA	0.008	0.022	-0.038 to 0.053	0.73318600
Group 3 (Lenke 3-4-6)	Time to first assisted ambulation (days)	Quadratus Lumborum FCSA	0.167	0.079	0.006 to 0.329	0.04260920
Group 3 (Lenke 3-4-6)	Time to first assisted ambulation (days)	Quadratus Lumborum Modified Goutallier Classification	-0.029	0.308	-0.657 to 0.599	0.92505100
Group 3 (Lenke 3-4-6)	Time to first assisted ambulation (days)	Quadratus Lumborum FA	0.201	0.529	-0.878 to 1.279	0.70697900
